# Intracellular acidity impedes KCa3.1 activation by Riluzole and SKA-31

**DOI:** 10.3389/fphar.2024.1380655

**Published:** 2024-04-04

**Authors:** Marco Cozzolino, Gyorgy Panyi

**Affiliations:** Department of Biophysics and Cell Biology, Faculty of Medicine, University of Debrecen, Debrecen, Hungary

**Keywords:** ion channels, KCa3.1, Riluzole, SKA-31, acidity, cancer

## Abstract

**Background:**

The unique microenvironment in tumors inhibits the normal functioning of tumor-infiltrating lymphocytes, leading to immune evasion and cancer progression. Over-activation of KCa3.1 using positive modulators has been proposed to rescue the anti-tumor response. One of the key characteristics of the tumor microenvironment is extracellular acidity. Herein, we analyzed how intra- and extracellular pH affects K^+^ currents through KCa3.1 and if the potency of two of its positive modulators, Riluzole and SKA-31, is pH sensitive.

**Methods:**

Whole-cell patch-clamp was used to measure KCa3.1 currents either in activated human peripheral lymphocytes or in CHO cells transiently transfected with either the H192A mutant or wild-type hKCa3.1 in combination with T79D-Calmodulin, or with KCa2.2.

**Results:**

We found that changes in the intra- and extracellular pH minimally influenced the KCa3.1-mediated K^+^ current. Extracellular pH, in the range of 6.0–8.0, does not interfere with the capacity of Riluzole and SKA-31 to robustly activate the K^+^ currents through KCa3.1. Contrariwise, an acidic intracellular solution causes a slow, but irreversible loss of potency of both the activators. Using different protocols of perfusion and depolarization we demonstrated that the loss of potency is strictly time and pH-dependent and that this peculiar effect can be observed with a structurally similar channel KCa2.2. While two different point mutations of both KCa3.1 (H192A) and its associated protein Calmodulin (T79D) do not limit the effect of acidity, increasing the cytosolic Ca^2+^ concentration to saturating levels eliminated the loss-of-potency phenotype.

**Conclusion:**

Based on our data we conclude that KCa3.1 currents are not sensitive the either the intracellular or the extracellular pH in the physiological and pathophysiological range. However, intracellular acidosis in T cells residing in the tumor microenvironment could hinder the potentiating effect of KCa3.1 positive modulators administered to boost their activity. Further research is warranted both to clarify the molecular interactions between the modulators and KCa3.1 at different intracellular pH conditions and to define whether this loss of potency can be observed in cancer models as well.

## 1 Introduction

Cytotoxic CD8^+^ T lymphocytes, in the front line of anti-cancer immunity, must migrate into the tumor to recognize and eliminate cancer cells. In addition to multiple intracellular signaling pathways that are involved in this process, it turned out that plasma membrane ion channels play an essential role in the regulation, migration and activation of T cells. Among other channels, the *Shaker*-related voltage-gated K^+^ channel Kv1.3, the calcium activated and intermediate conductance K^+^ channel KCa3.1 and the calcium-release-activated Ca^2+^ channel CRAC are implicated in these processes (Cahalan and Chandy, 2009). The ratio of the number of Kv1.3 to KCa3.1 channels in T cells depends on the phenotype that the T cells acquire after their activation (Cahalan and Chandy, 2009). The operation of the Kv1.3 and KCa3.1 channels is crucial for the maintenance of a permissive membrane potential for efficient Ca^2+^ signaling required for T cell activation and proliferation (Cahalan and Chandy, 2009), Chirra et al., reviewed and thoroughly analyzed recently how much the activity of K^+^ channels influence the survival of T cells, their cytokine production and their motility (Chirra et al., 2022).

Solid cancers, characterized by hypoxia and nutrient deprivation (Ahmadiankia et al., 2019), accumulate adenosine in the tumor microenvironment (TME) because of massive necrosis. There is evidence of a direct link between extracellular increase in the adenosine concentration and the progressive loss of functionality of KCa3.1 in infiltrating T cells: a deficit in KCa3.1 activity in CD8^+^ T cells hampers their ability to produce IL-2 and IFN-γ (Chimote et al., 2013) and inhibits their movement in response to chemotactic stimuli (Chimote et al., 2018). For similar reasons tumors are also rich in extracellular K^+^, which has been proved to cause an overall intracellular ionic impairment in local T cells, contributing to the suppression of the production of anti-tumoral cytokines (Eil et al., 2016). In both cases, a genetic over-expression of Kv1.3 (Chimote et al., 2013) or the pharmacologically induced activation of KCa3.1 (Chimote et al., 2013; 2020) re-established the normal functions of T cells.

In addition, the TME is also characterized by a severe disfunction of the pH homeostasis (Song et al., 1999; Damaghi et al., 2013). Hypoxia and nutrient deprivation have an impact on the metabolism of cancer cells triggering what is defined as the “Warburg effect,” which consists in the over-activation of the glycolytic pathway and the consequent accumulation of lactate and H^+^ in the extracellular milieu (Vaupel and Multhoff, 2021). The extracellular pH around the cancer cells can easily drop to 6.5–6.9 (Kato et al., 2018) and their intracellular pH rise to 7.3–7.6 (White et al., 2017) reversing the usual pH gradient across the cytoplasm membrane (from pH_e_ > pH_i_ to pH_e_ < pH_i_, where pH_e_ and pH_i_ are the extra- and intracellular pH, respectively) (Pérez-Herrero and Fernández-Medarde, 2021). Pancreatic ductal adenocarcinoma (PDAC), for example, because of its unique epithelia, has been recently at the center of attention in regard to its acidity (Pedersen et al., 2017). PET/MR showed that pH_e_ can decrease up to 6.4 after 7 days of PDAC growth in tumor-bearing mice (Goldenberg et al., 2018) and this can be even exploited to visualize the tumor itself and its metastases (Cruz-Monserrate et al., 2014). Moreover, the pH_e_ and the pH_i_ are intertwined and a change in the former has a direct effect on the latter (Michl et al., 2019). For example, it has been observed that an acidic extracellular medium tends to acidify the intracellular pH of human lymphocytes (Bjernertoth G et al., 1994; Erra Díaz et al., 2018; Navarro et al., 2022) which, as a consequence of acidosis, become dysfunctional and anergic (Calcinotto et al., 2012; Wu et al., 2019). Strikingly, a long-term exposure of CD8^+^ T-cells to a low pH caused the opposite effect, triggering their cell stemness and improving their anti-tumor efficacy (Cheng et al., 2023).

The extracellular pH is known to regulate the operation of several voltage-gated ion channels (Tombaugh and Somjen, 1996). This arises partially due to non-specific membrane surface charge screening effect and specific interactions with titratable amino acid side chains (Somodi et al., 2004). Extracellular acidic environment inhibits the opening of Kv1.3 by moving the activation threshold from −50 mV to more depolarized membrane potentials and slowing both activation and inactivation kinetics (Deutsch and Lee, 1989). Just like extracellular pH, intracellular pH influences ion channels as well. With regard to Kv1.3, intracellular acidification significantly dampens the K^+^ currents without affecting the kinetics and the activation threshold of the channel (Deutsch and Lee, 1989). Although KCa3.1 plays an important role in the function of CD8^+^ T cells as well (Chimote et al., 2018), its sensitivity to pH_e_ and pH_i_ has been not explored.

There are several molecules that enhance the current through KCa3.1 (Lin et al., 2022). The oldest positive modulator is 1-EBIO (Devor et al., 1996), but several other, stronger activators have thereafter been developed (Christophersen and Wulff, 2015). Since activation of K^+^ currents boosts T cells and counteracts cancer-induced immunological anergy (Eil et al., 2016; Chimote et al., 2018), their role as potential pharmacological tools to enhance the immune system in the TME has been suggested by Chandy and Norton (Chandy and Norton, 2016). On the other hand, it is not known if the potency of the KCa3.1 activators would be influenced by the pH_e_ and/or the pH_i_.

In this paper we aimed at characterizing the sensitivity of KCa3.1 currents both to intracellular and extracellular pH using whole-cell patch-clamp. Since KCa3.1 activators have been proposed to boost the anergic cancer-infiltrating T cells, it must be known whether the surrounding acidic microenvironment would hinder the effect of these drugs. To this end, we aimed and clarifying whether the potency of two of the common KCa3.1 activators (Riluzole and SKA-31) are influenced by pH_e_ and pH_i_.

## 2 Materials ad methods

### 2.1 Cell culture

Human venous blood from anonymized healthy donors was obtained from a blood bank. The peripheral blood lymphocytes (PBLs) were isolated through Histopaque1077 (Sigma-Aldrich Hungary, Budapest, Hungary) density gradient centrifugation. Cells obtained were resuspended in RPMI 1640 medium containing 10% fetal calf serum (Sigma-Aldrich), 100 μg/mL penicillin, 100 μg/mL streptomycin, and 2 mM L-glutamine, seeded in a 24-well culture plate at a density of 5 × 10^5^ cells per mL, and grown in a 5% CO_2_ incubator at 37°C for 2–5 days. Phytohemagglutinin A (PHA, Sigma-Aldrich) was added in 5, 7 or 10 μg/mL concentrations to the medium to boost the K^+^ channel expression.

Chinese hamster ovary (CHO) cells (gift from Yosef Yarden, Weizmann Institute of Science, Rehovot, Israel) were maintained by culturing in Dulbecco’s modified Eagle medium (DMEM, Gibco) supplemented with 2 mM L-glutamine, 10% FBS, 100 μg/mL streptomycin and 100 U/mL penicillin-G (Sigma-Aldrich) at a density of 0.5–1 × 10^6^ cells per mL in a humidified incubator at 37°C and 5% CO_2_. Cells were passaged 3 times in a week following a 2–5 min incubation in 0.05% trypsin-EDTA solution at 37°C. Cultures were used up to passage number 20. PCR-based tests were routinely used to detect *mycoplasma* infection, only mycoplasma-free cultures were used for experiments.

CHO cells that do not express endogenous voltage-gated ion currents (Voros et al., 2018) were transiently transfected with the following plasmids encoding hKCa3.1 and turboGFP in a pCMV6-AC-GFP (OriGene Technologies) vector; H192A-hKCa3.1 in a pEGFP-C1 vector (a kind gift from Bernard Attali, Tel Aviv University, Israel); hKCa2.2 in a pCDN3 plasmid (a kind gift from Bernard Attali, Tel Aviv University, Israel) and T79D-rCaM in a pcDNA3 plasmid (a kind gift from Bernard Attali, Tel Aviv University, Israel). TurboGFP is a modified version of ppluGFP2, derived from *Pontellina plumate*, and is characterized by a fluorescence up to three times higher than EGFP (Evdokimov et al., 2006). Transfections were performed using the Lipofectamine 2000 kit (Invitrogen, Carlsbad, CA) following the manufacturer’s protocol. The cells were grown under standard conditions (see CHO cell culturing). GFP-positive transfectants were identified using Nikon TMS fluorescence microscope (Nikon, Tokyo, Japan), and currents were recorded 24–48 h post transfection.

### 2.2 Electrophysiology and pharmacology

Electrophysiology measurements were carried out using the patch-clamp technique in voltage-clamp mode. Whole-cell currents were recorded from peripheral blood lymphocytes and transfected CHO cells using a Multiclamp 700B amplifier connected to a DigiData 1440A digitizer (Molecular Devices, Sunnyvale, CA, United States). Micropipettes were pulled from GC 150 F-15 borosilicate capillaries (Harvard Apparatus Kent, UK) resulting in 3–5 MΩ resistance in the bath solution. Current traces were lowpass-filtered through the built-in analog 4-pole Bessel filters of the amplifiers and sampling frequency was set at least twice the filter cutoff frequency. Recordings were carried out at room temperature (20°C–25°C), The patch-clamped cell was perfused with control and test solutions using a gravity-driven custom-built perfusion system and excess bath solution was removed constantly by vacuum suction. The cells were held at −85 mV holding potential to minimize the holding current and allow the calculation of the leak conductance. 150-ms-long voltage ramps (from −120 to +50 mV, in 150 ms) were applied every 10 s to evoke the KCa3.1 currents. The pClamp 10.5, 10.7, and 11.2 software packages were used to acquire the data.

### 2.3 Data analysis

For offline leak correction and the calculation of the K^+^ conductance a Python based custom written program was used (available upon request). The algorithm averaged the holding current (I_hold_) for *n* = 600 data points at −85 mV, which is close to the K^+^ equilibrium potential calculated from the Nernst equation (E_K_ = −89 mV). I_hold_ was considered as leak current and used to calculate G_leak_ as I_hold_/–85 mV. Every data point was corrected for linear leak using I_K_ = I_m_ – (G_leak_ × E_m_), where I_K_ is the leak subtracted K^+^ current, I_m_ is the measured membrane current at E_m_ membrane potential. I_K_ was then displayed as a function of E_m_ during the ramp and the region for linear current-voltage relationship was selected and G_K_ was calculated as the slope of the straight line fitted to the I_K_ data points (G_K_ = ΔI_K_/ΔV). The linear region was typically between −120 mV and −60 mV for lymphocytes, at more depolarized membrane potentials the activation of the Kv1.3 current caused a significant deflection from the linear I-V relationship.

The following parameters were derived from the leak subtracted G_K_ data:

G_K,200_/G_K,0_: ratio of the K^+^ conductances determined at a time point t >200 s (G_K,200_) in S-ECS over G_K_ at the beginning of the experiment (0 s) in S-ECS (G_K,0_) in the absence of KCa3.1 activators; G_K,x_/G_K,7.4_: normalized conductance, G_K_ values determined in various pH_e_ solutions (G_K,x_, where x is the pH_e_) in a given cell normalized to G_K,7.4_ recorded at pH_e_ = 7.4 in the same cell; G_K,act_/G_K_: “fold increase in conductance,” K^+^ conductance measured in the presence of the activator at a given concentration (G_K,act_) divided by the K^+^ conductance in the drug-free solution (G_K_) “fold increase normalized to 7.4”: fold increase in conductance” caused by the activator at a given pH_i_ and pH_e_ combination normalized to the “fold increase in conductance” measured with the activators in S- ECS at pH_e_ = 7.4.

G_K,end_/G_K,start_: G_K,end_ and G_K,start_ are the activator-enhanced K^+^ conductance at the end of the experiment (≥800 s) and the activator-enhanced K^+^ conductance at the beginning of the experiment, respectively.

The activator concentration-response curves were fit using:

$$\text{fold increase in conductance}=\text{Bottom}+\frac{\left(Top-Bottom\right)\times {\left[X\right]}^{n_{H}}}{{\left[X\right]}^{n_{H}}+{{EC}_{50}}^{n_{H}}}$$

where “fold increase in conductance” is defined above, [X] is the activator concentration, EC_50_ is the concentration of agonist that gives a response halfway between Bottom (min value of “fold increase in the conductance”) and Top (maximum value of the “fold increase in conductance”), and n_H_ is the Hill slope. The Clampfit 10.7 and 11.1 software packages (Molecular Devices Inc., Sunnyvale, CA) were used to analyze the data and subtract the leak currents in the pilot experiments. Statistical analyses were performed with GraphPad Prism 8.4.3 (GraphPad Software, Inc., San Diego, CA).

### 2.4 Solutions

The standard extracellular solution (S-ECS) was a Na^+^-aspartate (Na^+^Asp^−^)-based solution with 2.5 mM CaCl_2_, and 10 mM HEPES titrated to pH_e_ = 7.4 with NaOH. The extracellular solution having pH_e_ = 8.0 (8.0-ECS) was buffered with 10 mM HEPES whereas those having pH_e_ = 6.0 (6.0-ECS); pH_e_ = 6.5 (6.5-ECS) or pH_e_ = 6.9 (6.9-ECS) were buffered using 10 mM MES, pH was titrated to the desired value with NaOH (see Table 1 for the composition of the solutions). The standard intracellular solution (S-ICS) was a K^+^-aspartate (K^+^Asp^−^)-based solution with 8.5 mM CaCl_2_, 10 mM EGTA and 10 mM HEPES (titrated to pH_i_ = 7.2 with Tris). This solution has an estimated free Ca^2+^ concentration of ∼1-2 μM based on the MaxChelator program WEBMAX-C software (C. Patton, Stanford University, retrieved here: https://somapp.ucdmc.ucdavis.edu/pharmacology/bers/maxchelator/webmaxc/webmaxcE.htm). Since pH has a strong effect on the affinity of EGTA for Ca^2+^ we substituted EGTA with BAPTA, a less pH-sensitive Ca^2+^ chelator (Bers et al., 2010), when the pH of the intracellular solution was titrated to various levels. To keep the free Ca^2+^ concentration around ∼1-2 μM the pipette-filing solution titrated to pH_i_ = 6.5 (6.5-ICS) contained 11 mM BAPTA whereas the one titrated to pH_i_ = 8.0 (8.0-ICS) contained 10 mM BAPTA. For the experiments in which we used the KCa3.1 activators at pH = 7.2 we set the free Ca^2+^ concentration in the pipette-filing solution to ∼250 nM (7.2-ICS-250), as suggested by Jenkins et al. (2013) and Ca^2+^ was buffered using EGTA (10 mM EGTA and 5.7 mM CaCl_2_). When the pH of the pipette-filling solution, with 250 nM Ca^2+^ concentration, was set to pH_i_ = 6.5 (6.5-ICS-250) or pH_i_ = 8.0 (8.0-ICS-250) the amount of BAPTA was set to 10 mM, but the concentration of CaCl_2_ was adjusted to reflect the pH-dependence of the buffer capacity of BAPTA. More precisely, in case of pH_i_ = 6.5 we used 3.25 mM CaCl_2_ and for pH_i_ = 8.0 we used 4.3 mM. All pipette-filling solutions were titrated with Tris. All titrations were done at 25°C and the pH of solutions was checked before every experiment.

**TABLE 1:** Composition of the intra- and extracellular solutions.

	S-ECS, 8.0-ECS, 6.9-ECS, 6.5-ECS, 6.0-ECS	S-ICS	8.0-ICS	6.5-ICS	7.2-ICS-250	8.0-ICS-250	6.5-ICS-250
Na^+^Asp^-^	145	—	—	—	—	—	—
K^+^Asp^-^	—	145	145	145	145	145	145
KCl	5	—	—	—	—	—	—
MgCl_2_	1	2	2	2	2	2	2
CaCl_2_	2.5	8.5	8.5	8.5	5.7	4.3	3.25
Glucose	5.5	—	—	—	—	—	—
HEPES	10^*^	10	10	-	10	10	-
MES	10^**^	—	—	10	—	—	10
EGTA	—	10	-	-	10	-	-
BAPTA	—	—	10	11	—	10	10
pH	6.0/6.5/6.9/7.4/8.0 (w/NaOH)	7.2 (w/Tris)	8.0 (w/Tris)	6.5 (w/Tris)	7.2 (w/Tris)	8.0 (w/Tris)	6.5 (w/Tris)
Free Ca^2+^	(2.5 mM)	∼1 μM	∼1–2 μM	∼1–2 μM	∼250 nM	∼250 nM	∼250 nM

The numbers in the cells are the concentrations of the solutes in mM. Asp^−^: aspartate; ECS, extracellular solution; ICS, pipette-filling intracellular solution. Numbers preceding the ECS/ICS designation indicate the pH, following the ECS/ICS designation, when applicable, indicate the free Ca^2+^ concentration (i.e. 7.2-ICS-250 means pipette-filling intracellular solution at pH = 7.2 and free Ca^2+^ concentration of 250 nM). *: HEPES, buffer was used for pH = 7.4 and pH = 8.0; ** MES buffer was used for pH = 6.0, pH = 6.5 and pH = 6.9.

### 2.5 Chemicals

All salts and components of the solutions were purchased from Sigma-Aldrich Budapest, Hungary. The KCa3.1 activators Riluzole (6-(trifluoromethoxy) benzothiazol-2-amine) and SKA-31 (Naphtho [1,2-d]thiazol-2-ylamine) and the blocker TRAM-34 (1-[(2-chlorophenyl)-di (phenyl) methyl] pyrazole), a gentle gift by prof. Heike Wulff, were kept in DMSO at a stock concentration of 5–10 mM and suitably diluted in the extracellular solutions (see Table 1) as needed.

## 3 Results

### 3.1 Extra- and intracellular pH-dependence of whole-cell KCa3.1 currents in human T cells

**FIGURE 1:**
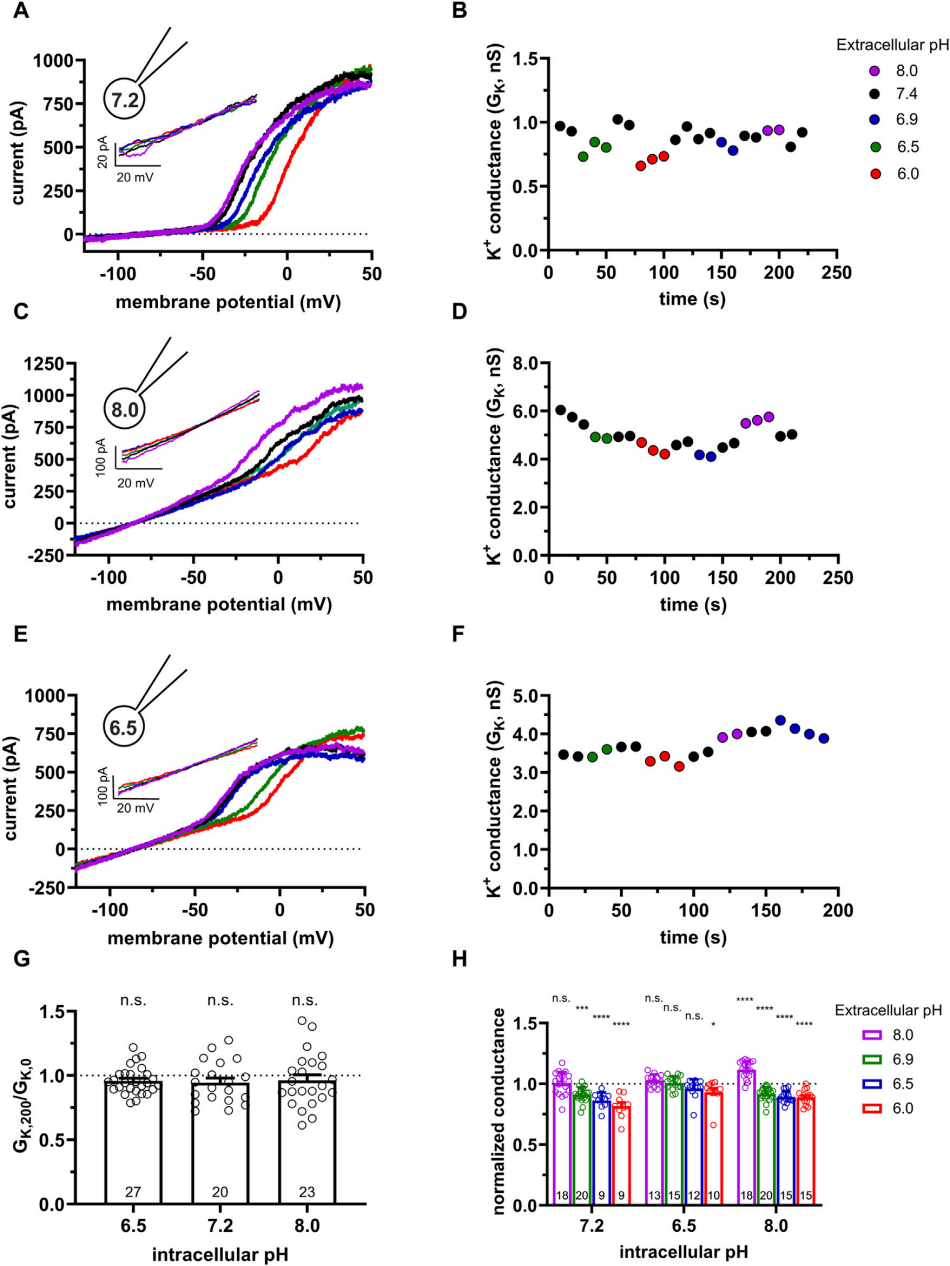
Effect of the extra- and intracellular pH on KCa3.1 currents in human PBLs. **(A,C,E)** Representative current traces were evoked by 150-ms-long voltage ramps, ranging from −120 to +50 mV in whole-cell patch-clamped human peripheral T cells. Voltage ramps were repeated every 10 s, the holding potential was −85 mV between pulses. The pipette filling solutions were S-ICS (pH_i_ = 7.2, panel A), 8.0-ICS (pH_i_ = 8.0, panel C), and 6.5-ICS (pH_i_ = 6.5, panel E), the cells were perfused with extracellular solutions having pH_e_ = 6.0 (6.0-ECS, red), pH_e_ = 6.5 (6.5-ECS, blue), pH_e_ = 6.9 (6.9-ECS, green), pH_e_ = 7.4 (S-ECS, black), and pH_e_ = 8.0 (8.0-ECS, purple). Insets show the KCa3.1-specific K^+^ current measured below the activation threshold of Kv1.3, between −120 mV and −60 mV. **(B,D,F)** KCa3.1-specific K^+^ conductance (G_K_) was determined by fitting straight lines to the traces in the insets in panels A, C, and E and plotted as a function of time for pH_i_ = 7.2 (B, same cell as in A), pH_i_ = 8 (D, same cell as in C) and pH_i_ = 6.5 (F, same cell as in E). The color of the symbols indicates the pH of the extracellular solutions. **(G)** The ratio of the K^+^ conductances determined at t > 200 s (G_K,200_) in S-ECS over G_K_ at the beginning of the experiment (t = 0 s) in S-ECS (G_K,0_), indicated as G_K,200_/G_K,0_. The pH_i_ of the pipette-filling solutions is indicated. Bars and error bars indicate the mean ± SEM, symbols show individual values, and numbers in the bars indicate the number of cells. **(H)** Normalized K^+^ conductance was calculated as G_K,x_/G_K,7.4_, where G_K,x_ and G_K,7.4_ are the KCa3.1-specific K^+^ conductances at pH_e_ = x and pH_e_ = 7.4, respectively. Bars are grouped by the pH of the pipette-filling solution (pH_i_). Bars and error bars indicate the mean ± SEM, symbols show individual values, numbers in the bars indicate the number of cells. Statistical analysis **(G,H)** was performed using one-way ANOVA (against H_0_:μ_0_ = 1 hypothesis) with multiple comparisons (Bonferroni). *p < 0.05, ***p < 0.001, ****p < 0.0001, n.s., not significant (*p* > 0.05). Extracellular pH was represented in all cases with the same colors: purple for 8.0, black for 7.4, green for 6.9, blue for 6.5, and red for 6.0.

The number of KCa3.1 channels in resting T cells is ∼10 (Cahalan and Chandy, 2009) and considering the ∼11–40 pS conductance of the channel (Aldrich et al., 2023), the magnitude of the whole-cell current is too small for precise pharmacological experiments. The expression of KCa3.1 is transcriptionally upregulated when T cells are stimulated with mitogens (Ghanshani et al., 2000; Wulf and Schwab, 2002). We took advantage of this and stimulated T cell proliferation using phytohemagglutinin A (PHA) to increase KCa3.1 expression. In addition to PHA activation of the T cells we also used 1 µM Ca^2+^ concentration in the pipette filling solution to fully activate the KCa3.1 current (Sforna et al., 2018). PHA-treatment also upregulates the expression of the voltage-gated Kv1.3 K^+^ channel. One can record isolated KCa3.1 currents in activated T cells using either the pharmacological separation, i.e., blockage of the Kv1.3 current using peptide toxins (Varga et al., 2021), or analyze the whole-cell currents below the activation threshold of Kv1.3. We chose the second scenario to avoid the use of multiple pharmacological tools when KCa3.1 activators were studied. The records in Figure 1A were obtained in a whole-cell patch-clamped T cell at several extracellular pH values during voltage ramps ranging from −120 mV to +50 mV. The records can be split on the voltage axis into two regions: a linear part between −120 mV and −50 mV and a very strongly outwardly rectifying part corresponding to the voltage-dependent activation of Kv1.3 at membrane potentials more positive than −50 mV (Panyi et al., 2006). Consequently, we have assigned the currents to KCa3.1 in the region between −120 mV and −60 mV (see inset to Figure 1A). The inset shows clearly that the current-voltage relationship is linear in this range and thus, the slope of the straight lines fitted to the highlighted part of the traces (inset) were used to determine the KCa3.1-specific K^+^ conductance and were denoted as G_K_ thereafter (calculated as G_K_ = ΔI/ΔV). The reversal potential (E_rev_) of the leak-corrected currents is between −75 mV and −100 mV which indicates the K^+^ selectivity of the current (theoretical E_rev_ of a K^+^ selective conductance calculated from the Nernst equation is E_K_ = −89 mV). The reversal potential of the current being close to the theoretical equilibrium potential for K^+^ is a strong indicator for the quality of the records (i.e., small contamination from leak). Thus, we used G_K_ to assess quantitatively the magnitude of the KCa3.1 current as the K^+^ gradient remained constant throughout the experiments. Moreover, we used TRAM-34 to confirm that the current between −120 mV and −60 mV is KCa3.1 (Supplementary Figure S1) The slope of the current is reduced substantially upon perfusion the recording chamber with S-ECS containing 20 nM TRAM-34, which is a high affinity and specific inhibitor of KCa3.1 (IC_50_ = 20 nM, (Wulff et al., 2000), Supplementary Figure S1A, inset). The G_K_ determined from the slopes decreased gradually following the start of the perfusion (Supplementary Figure S1B) with TRAM-34. We used TRAM-34 (20 nM) routinely at the end of the experiments to confirm that the currents between −120 mV and −60 mV were KCa3.1-specific.

Figure 1A also shows that the activation threshold of Kv1.3 becomes progressively more positive as the extracellular pH decreases from pH_e_ = 8.0 to pH_e_ = 6.0. This is consistent with the pH_e_-dependence of the activation threshold for Kv1.3 reported earlier (Deutsch and Lee, 1989). Figure 1A and the inset shows qualitatively that the KCa3.1 currents recorded at pH_e_ values ranging from pH_e_ = 8.0 to pH_e_ = 6.0 are superimposable when the S-ICS (pH = 7.2) was used as the pipette filling solution. Figure 1B shows the G_K_ obtained at different pH_e_ values with S-ICS in the pipette (in the same experiment as in A). The G_K_ values were consecutively determined when the recording chamber was perfused with extracellular solutions having five different pH_e_ values. The perfusion started with the pH_e_ = 7.4 solution (S-ECS) as the reference solution and then sequentially switched to a different pH_e_ solutions, as indicated, and back to the pH_e_ = 7.4 solution. Figure 1B indicates that changing of pH_e_ alters minimally but fully and readily reversibly G_K_. Data in Figure 1A was obtained using the standard pipette-filling solution of pH_i_ = 7.2 (S-ICS). The same set of experiments as in Figures 1A, B were repeated using pipette-filling solution having pH_i_ = 8.0 (8.0-ICS) and pH_i_ = 6.5 (6.5-ICS) (Figures 1C–F). The results were essentially the same as at pH_i_ = 7.2: the raw currents below the activation threshold of Kv1.3 and recorded in different pH_e_ solutions are virtually superimposable (Figures 1C, E, and insets), the currents reverse negative to −75 mV and the G_K_ values are minimally sensitive to changing pH_e_ of the extracellular solution (Figures 1D, F). Moreover, G_K_ values at pH_e_ = 7.4 are relatively constant throughout a given experiment, regardless of the pH_i_. Taking the ratio of the K^+^ conductances determined at a time point t > 200 s (G_K,200_) in S-ECS over G_K_ at the beginning of the experiment (0 s) in S-ECS (G_K,0_) resulted in G_K,200_/G_K,0_ ∼1 thereby indicating the stability of the whole-cell hKCa3.1 currents (Figure 1G). The statistical analysis of the G_K_ values at all pH_e_ and pH_i_ combinations used in this study is in Figure 1H. G_K_ values determined in various pH_e_ solutions (G_K,x_) in a given cell were normalized to the ones recorded at pH_e_ = 7.4 in the same cell (normalized conductance = G_K,x_/G_K,7.4_) to allow the comparison of the data obtained in different cells. The bar graph in Figure 1H shows that at pH_i_ = 7.2 and pH_i_ = 8.0 there is a small, but significant decrease in the normalized conductance when the extracellular pH was changed to acidic ones, whereas the conductances were essentially the same when pH_i_ was 6.5 regardless of the pH_e_.

### 3.2 Extra- and intracellular pH-dependence of whole-cell KCa3.1 currents in CHO cells

**FIGURE 2:**
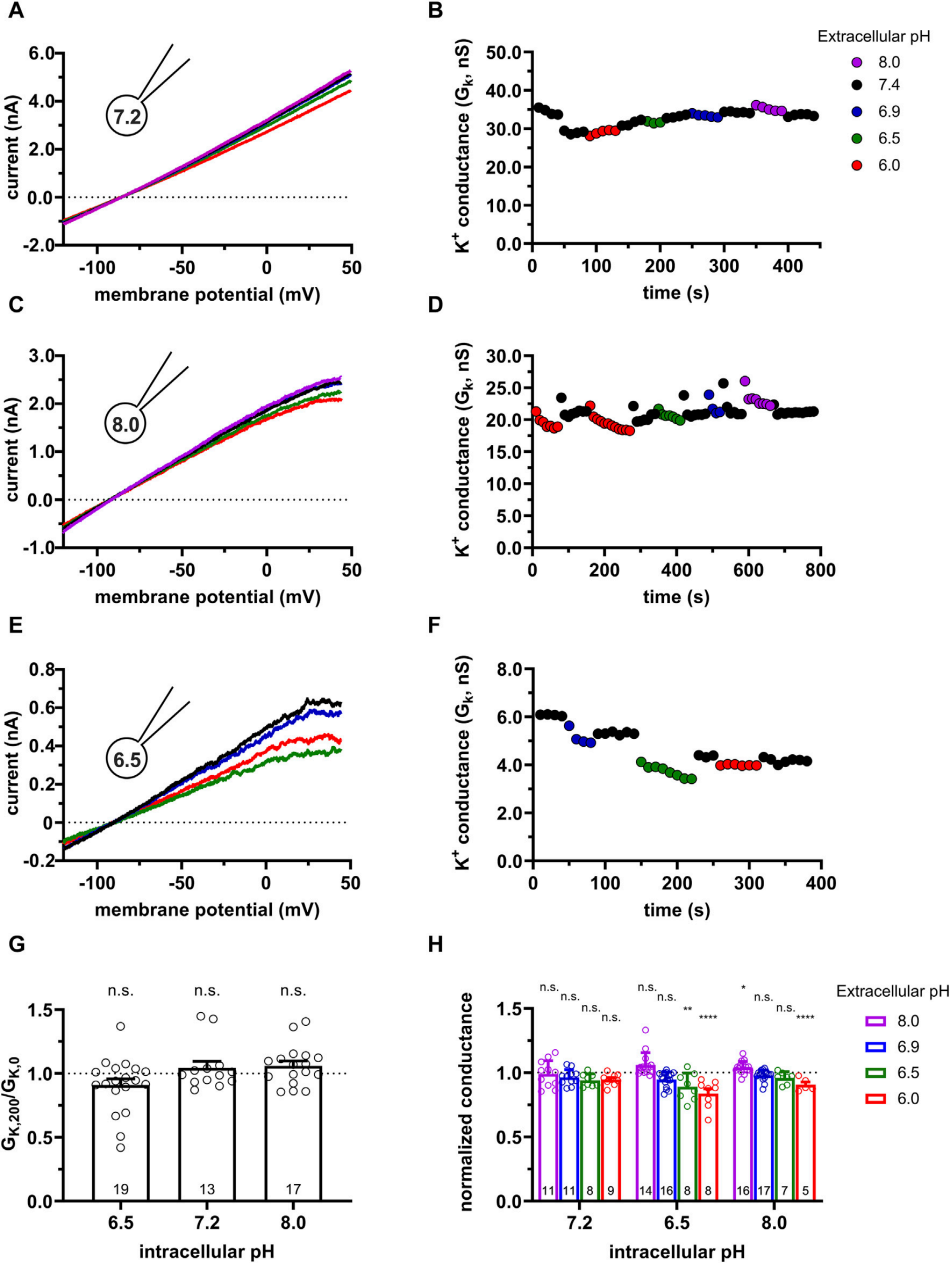
Effect of the extracellular and intracellular pH on currents generated in CHO cells transfected with turboGFP-hKCa3.1. **(A,C,E)** Representative current traces were evoked by 150-ms-long voltage ramps, ranging from −120 to +50 mV in whole-cell patch-clamped CHO cells expressing hKCa3.1 channels. Voltage ramps were repeated every 10 s, the holding potential was −85 mV between pulses. The pipette filling solutions were S-ICS (pH_i_ = 7.2, panel A), 8.0-ICS (pH_i_ = 8.0, panel C) and 6.5-ICS (pH_i_ = 6.5, panel E) and perfused with extracellular solutions having pH_e_ = 6.0 (6.0-ECS, red), pH_e_ = 6.5 (6.5-ECS, blue), pH_e_ = 6.9 (6.9-ECS, green), pH_e_ = 7.4 (S-ECS, black), and pH_e_ = 8.0 (8.0-ECS, purple). **(B,D,F)** K^+^ conductance (G_K_) was determined by fitting straight lines to the traces in panels A, C, and E and plotted as a function of time for pH_i_ = 7.2 (B, same cell as in A), pH_i_ = 8 (D, same cell as in C) and pH_i_ = 6.5 (F, same cell as in E). The color of the symbols indicates the pH of the extracellular solutions. **(G)** Ratio of the K^+^ conductances determined at t > 200 s (G_K,200_) in S-ECS over G_K_ at the beginning of the experiment (t = 0 s) in S-ECS (G_K,0_), indicated as G_K,200_/G_K,0_. The pH_i_ of the pipette-filling solutions are indicated. Bars and error bars indicate the mean ± SEM, symbols show individual values, numbers in the bars indicate the number of cells. **(H)** Normalized K^+^ conductance was calculated as G_K,x_/G_K,7.4_, where G_K,x_ and G_K,7.4_ are the K^+^ conductances at pH_e_ = x and pH_e_ = 7.4. Bars are grouped by the pH of the pipette-filling solution (pH_i_). Statistical analysis was performed using one-way ANOVA (against H_0_:μ_0_ = 1 hypothesis) with multiple comparison (Bonferroni) **(G,H)**. *p < 0.05, **p < 0.01, ****p < 0.0001, n.s., not significant (*p* > 0.05).

We repeated the same set of experiments as in Figure 1. Using cells transfected with the hKCa3.1 gene with the following motivations: 1) transfection can be optimized to increase KCa3.1 conductance therefore minimize the effect of leak on the whole-cell K^+^ conductance and 2) CHO cells do not express Kv1.3 (Yu and Kerchner, 1998), therefore one can minimize the contamination of the data by other K^+^ conductances. These become critical when the effect of the activators was studied (see below). Moreover, CHO can be transfected with mutant KCa3.1 and Calmodulin constructs (see below). Figure 2A shows the K^+^ current recorded in a CHO cell transfected with the hKCa3.1 gene that contains a turboGFP tag for the identification of the transfectants. Upon obtaining whole-cell configuration voltage ramps were applied and robust KCa3.1 currents (several nA at +50 mV) were recorded regardless of the extracellular pH. The current-voltage relationships are linear for voltages up to 0 mV and the reversal potential of the currents is more negative than −75 mV. Similarly to human lymphocytes, KCa3.1 currents recorded at pH_e_ values ranging from pH_e_ = 8.0 to pH_e_ = 6.0 in CHO cells are superimposable when the S-ICS (pH = 7.2) was used as the pipette filling solution. Figure 2B shows G_K_ obtained at different pH_e_ values (same experiment as in Figure 2A). The G_K_ values were consecutively determined when the recording chamber was perfused with extracellular solutions having five different pH_e_ values. Figure 2B indicates that changing of pH_e_ alters minimally but fully and readily reversibly G_K_. Moreover, G_K_ determined at pH_e_ = 7.4 is constant during the >200 s duration of the experiment. The same set of experiments as in Figures 2A, B were repeated using pipette-filling solution having pH_i_ = 8.0 (8.0-ICS, Figures 2C, D) and pH_i_ = 6.5 (6.5-ICS, Figures 2E, F). The current density measured at +50 mV was insensitive to the pH_i_ (Supplementary Figure S2). Figure 2G allows us to draw a similar conclusion to what was obtained for human peripheral blood lymphocytes, i.e., G_K_ values at pH_e_ = 7.4 are constant throughout a given experiment, regardless of the pH_i_. The statistical analysis of the G_K_ values at all pH_e_ and pH_i_ combinations were performed the same way as for human peripheral blood lymphocytes (see details above). The normalized conductance (G_K,x_/G_K,7.4_) values in the bar graph in Figure 2H show that at some pH_i_ and pH_e_ combinations there was a small, but significant decrease in the normalized conductance.

### 3.3 Concentration-dependence of the effect of the KCa3.1 activators

Riluzole and its more potent derivative SKA-31 (Sankaranarayanan et al., 2009) are positive modulators of the KCa3.1 currents in micro- and nanomolar-range concentrations, respectively. However, the pH-dependence of the action of these compounds has not been elucidated. To ensure that the applied concentration of the activators is not saturating (i.e., either loss or increase in their potency can be measured at different pH_i_-pH_e_ combinations) we have experimentally determined the EC_50_ values for Riluzole and SKA-31 in CHO cells. As the potentiation of the KCa3.1 current by the activators is more pronounced at low cytosolic Ca^2+^ concentrations as compared to ∼1 µM Ca^2+^ concentration used above (Figures 1, 2), we used pipette filling solutions having 250 nM Ca^2+^ concentration (7.2-ICS-250) in this set of experiments (Jenkins et al., 2013). The KCa3.1 currents in lymphocytes in 7.2-ICS-250 are very small and this means that the K^+^ conductance determined from the slopes of the voltage-ramps can be contaminated by leak. This becomes critical since the potency of the activators is expressed as fold change in the KCa3.1 conductance (Jenkins et al., 2013), where G_K_ in the absence of the activator is in the denominator. To overcome this, especially at low activator concentrations, we chose to obtain the EC_50_ values in CHO overexpressing KCa3.1 where G_K_ in the absence of the activators can be determined more precisely than in human peripheral blood lymphocytes.

**FIGURE 3:**
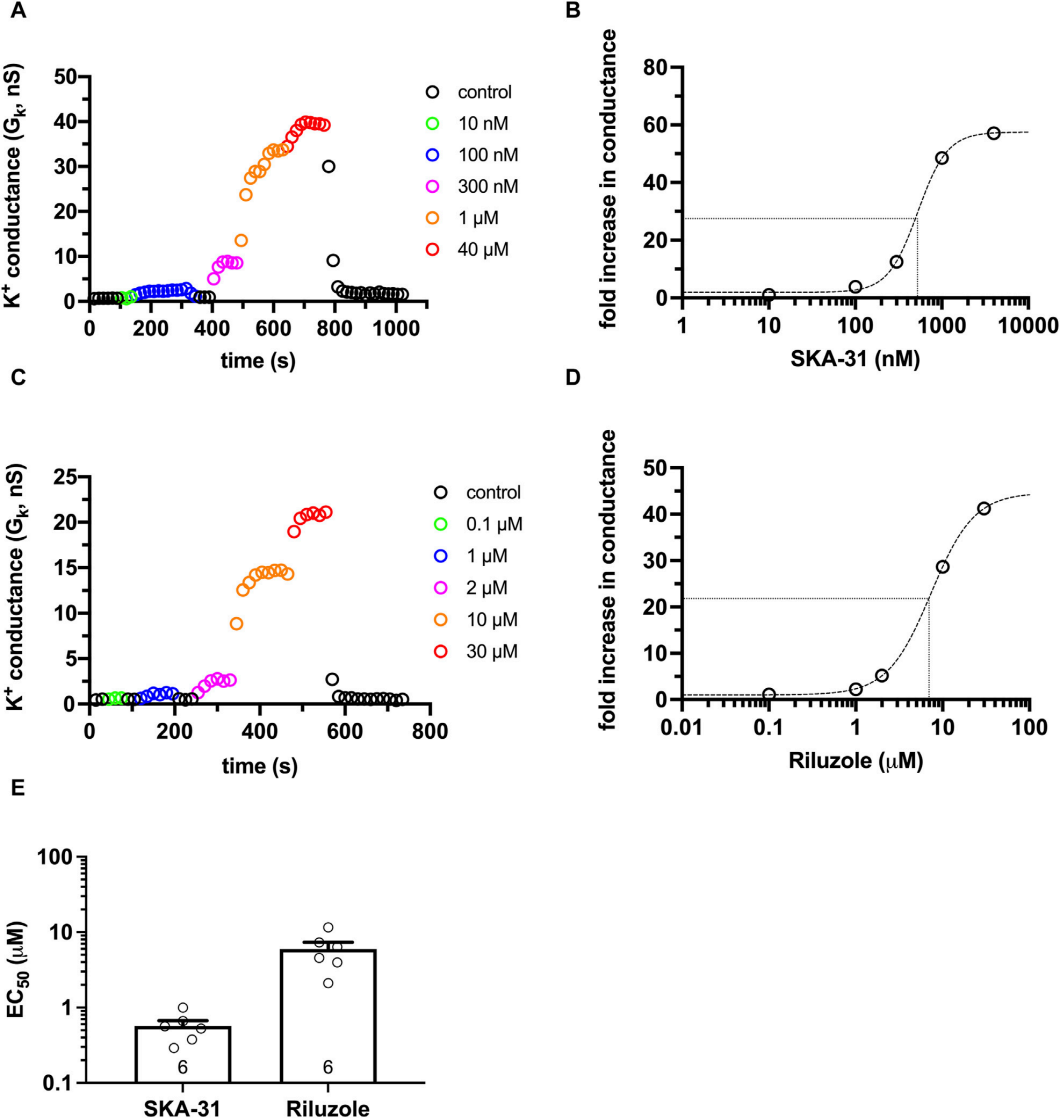
Activator concentration-response for SKA-31 and Riluzole in CHO. KCa3.1 currents were recorded in CHO cells transfected with turboGFP-hKCa3.1 using S-ECS bath solution (pH_e_ = 7.4) and 7.2−ICS−250 pipette filling solution (250 nM free Ca^2+^ concentration, pH_i_ = 7.2). The activators at the indicated concentrations were diluted in S-ECS. **(A)** KCa3.1 currents were evoked by voltage ramps ranging from −120 mV to +50 mV, K^+^ conductance was determined from the slope of the current-voltage relationship and plotted as a function of time. Colored symbols represent the indicated concentrations of SKA-31. **(B)** Activator concentration-response for SKA-31 for the cell shown in panel A. Fold increase in the conductance was calculated as G_K,act_/G_K,_ where G_K,act_ and G_K_ are the K^+^ conductance measured in the presence and the absence of the activator in S-ECS, respectively. The dashed line is the best-fit Hill equation with EC_50_ = 526.4 nM, n_H_ = 2.5. The half maximal activation and the EC_50_ are indicated by the dotted lines. **(C)** Same experiment as in panel A, except the indicated concentrations of Riluzole were used. **(D)** The activator concentration-response Riluzole for the cell shown in panel C, see details in panel B. EC_50_ = 7.37 µM, n_H_ = 1.74 were obtained. **(E)** EC_50_ values determined from fitting individual activator concentration-response relationships for Riluzole and SKA-31. Symbols in the bar graph (mean ± SEM) show individual values, and numbers in the bar indicate the number of cells.

Figure 3 shows representative activator concentration-response experiments for SKA-31 (Figure 3A) and for Riluzole (Figure 3C). The G_K_ values were determined from voltage-ramp experiments in control extracellular solution (S-ECS) and S-ECS solutions containing the activators in the indicated concentrations. Figures 3A, C show, especially at higher activator concentrations, that the K^+^ conductance increases drastically and rapidly upon perfusing the recording chamber with the activators and declines with rapid kinetics when the chamber is perfused with S-ECS. The activator concentration-response curves (Figures 3B, D) were constructed by calculating the “fold increase in conductance” by dividing the K^+^ conductance measured in the presence of the activator at a given concentration (G_K,act_) with the K^+^ conductance in the drug-free solution (G_K_), i.e., G_K,act_/G_K_. The best-fit Hill equations to the data points obtained for individual plots (Figures 3B, D) resulted in EC_50_ = 570 ± 101 nM (*n* = 6) for SKA-31 and 6.0 ± 1.3 μM (*n* = 6) for Riluzole (Figure 3E). Based on the EC_50_ values we chose 1 µM SKA-31 and 5 µM Riluzole concentrations where the fold increase in the G_K_ was 52.3 ± 10.4-fold (*n* = 8) for SKA-31 and between 4.5 (2 µM) and 23-fold (10 µM) for Riluzole. The highest concentration of SKA-31 tested in our experiments (4 µM) caused 79.3 ± 17.5-fold increase in G_K_ (*n* = 6), the same parameter for 30 µM Riluzole was 37.4 ± 17.9-fold increase (*n* = 5).

### 3.4 Effect of KCa3.1 activators as a function of intra- and extracellular pH in PBLs

**FIGURE 4:**
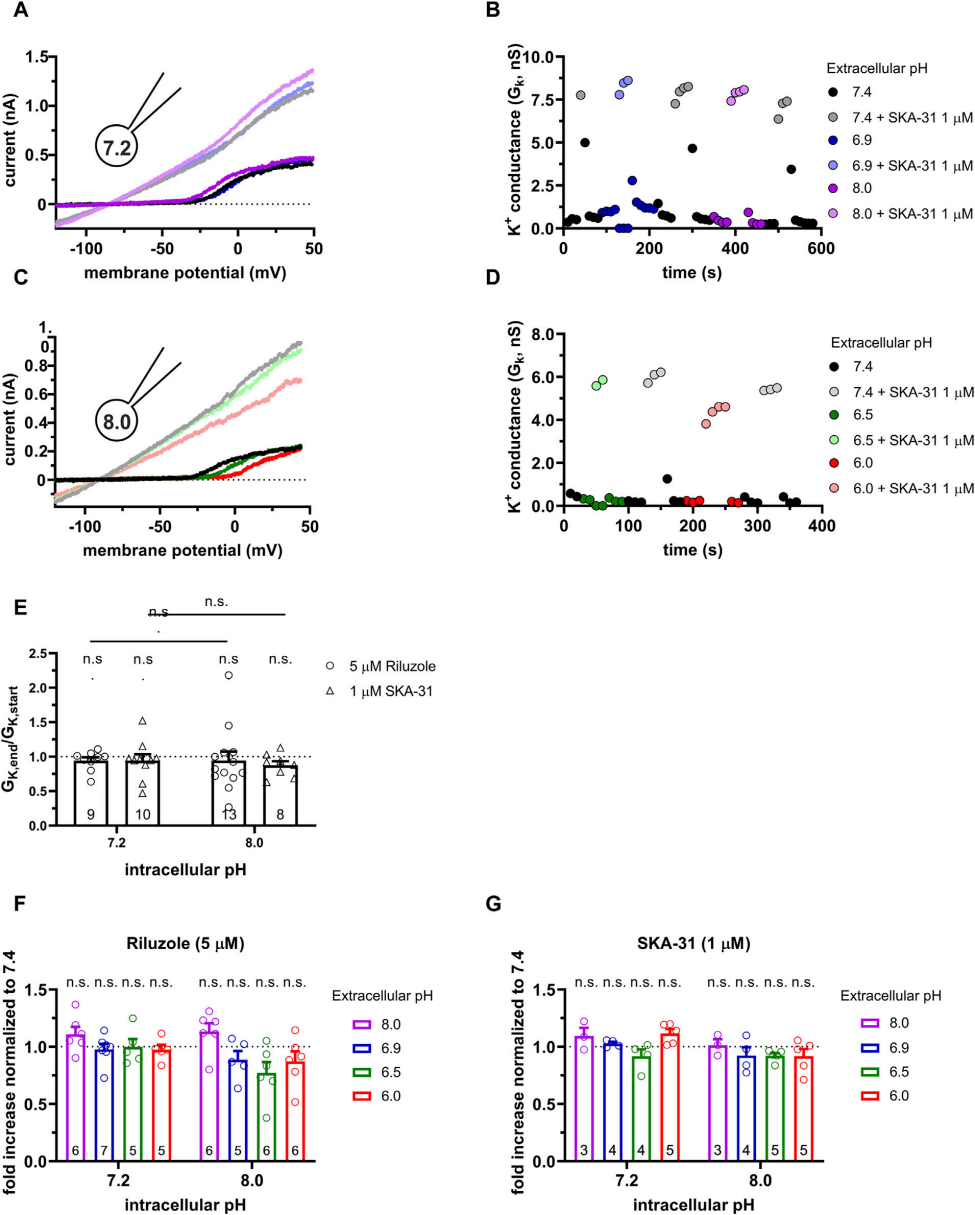
Effect of SKA-31 on the KCa3.1 currents in PHA-activated PBLs at different pH_e_ and pH_i_ combinations. **(A,C)** Representative current traces were evoked by 150-ms-long voltage ramps, ranging from −120 to +50 mV in whole-cell patch-clamped human peripheral T cells. Voltage ramps were repeated every 10 s, the holding potential was −85 mV between pulses. The pipette filling solutions were 7.2-ICS-250 (pH_i_ = 7.2, panels A and B) or 8.0-ICS-250 (pH_i_ = 8.0, panels C and D). The cells were perfused with extracellular solutions having pH_e_ = 6.0 (6.0-ECS, red), pH_e_ = 6.5 (6.5-ECS, blue), pH_e_ = 6.9 (6.9-ECS, green), pH_e_ = 7.4 (S-ECS, black), and pH_e_ = 8.0 (8.0-ECS, purple). The corresponding lighter colors display traces obtained in the presence of the modulator. **(B,D)** KCa3.1-specific K^+^ conductance (G_K_) was determined by fitting straight lines to the traces below the activation threshold of Kv1.3 in panels A, B and plotted as a function of time. B: Data from panel A: pH_i_ = 7.2. pH_e_ and 1 µM SKA-31 as indicated. D: Data from panel B: pH_i_ = 8.0, pH_e_ and 1 µM SKA-31 as indicated. **(E)** Loss of the potency of the modulators was expressed as G_K,end_/G_K,start_ ratio (G_K,end_ and G_K,start_ are the averaged K^+^ conductances with the presence of the activator in S-ECS at the end and at the beginning of the experiment, respectively), calculated for each cell and plotted as a bar graph (mean ± SEM). Symbols show individual values, numbers in the bar indicate the number of cells. **(F,G)** The “fold increase normalized to 7.4” was calculated dividing the activator-induced “fold increase in conductance” (see e.g., Figure 3B) at a given pH_i_ and pH_e_ combination by the “fold increase in conductance” measured with the activators in S-ECS at pH_e_ = 7.4. Statistical analysis was performed using one-way ANOVA (against H_0_:μ_0_ = 1 hypothesis) with multiple comparison (Bonferroni) **(E,F,G)** and Student’s unpaired *t*-test (Riluzole 7.2 vs. SKA-31 7.2, Riluzole 8.0 vs. SKA-31 8.0) **(E)**. n.s., not significant (*p* > 0.05).

**FIGURE 5:**
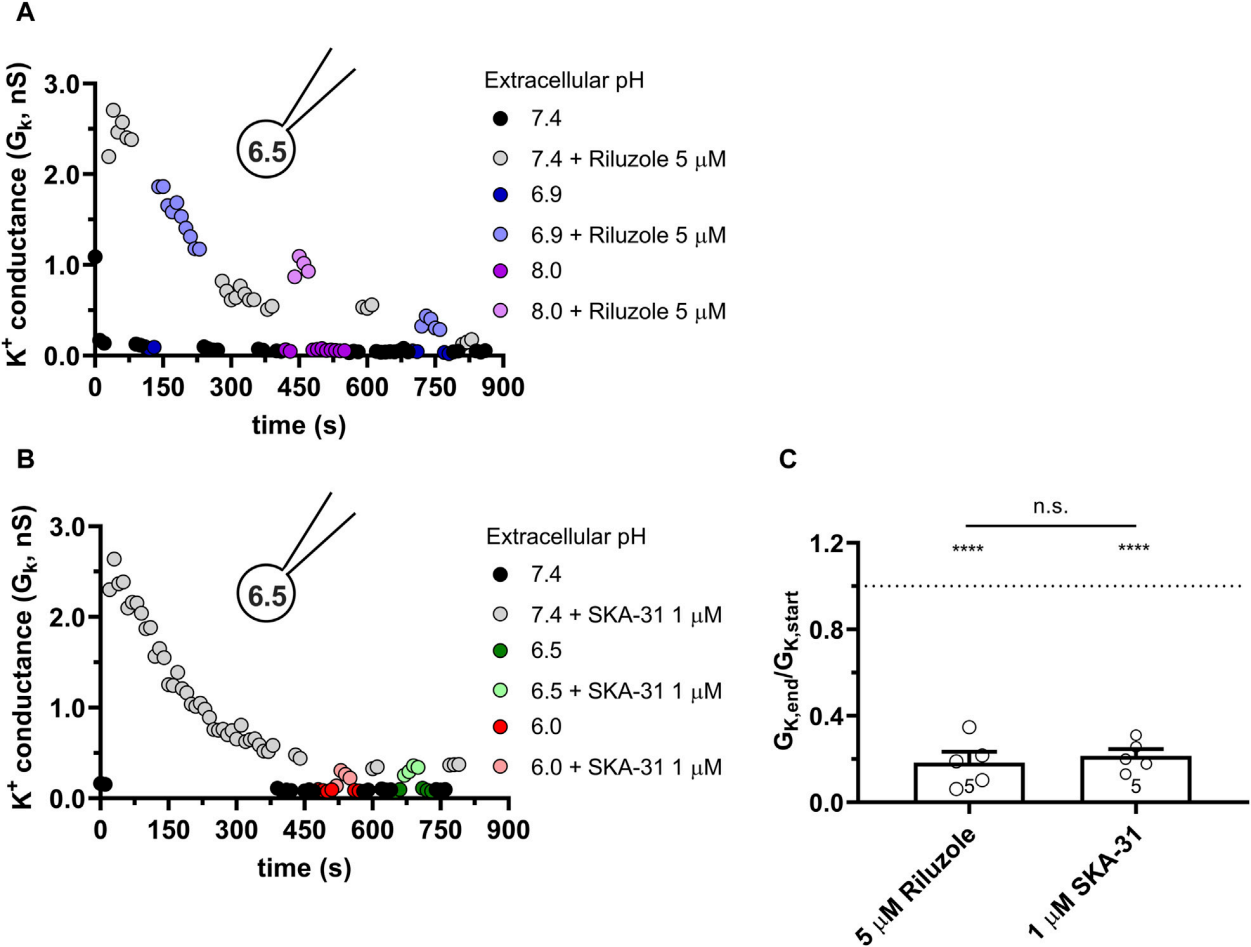
Progressive loss of the potency of Riluzole and SKA-31 in activating KCa3.1 currents of PBLs at intracellular acidic condition. Representative time courses of the effect of Riluzole **(A)** and SKA-31 **(B)** when the intracellular solution was kept at pH_i_ = 6.5 (6.5-ICS-250) and cells were perfused with extracellular solutions having different pH_e_ values (ranging from 6.0 to 8.0) and modulators (A: 5 µM Rilozole, B: 1 µM SKA-31), as indicated. The current traces were evoked by 150-ms-long voltage ramps, ranging from −120 to +50 mV in whole-cell patch-clamped human peripheral T cells. Voltage ramps were repeated every 10 s, the holding potential was −85 mV between pulses. **(C)** Loss of the potency of the modulators was expressed as G_K,end_/G_K,start_ ratio (G_K,end_ and G_K,start_ are the averaged K^+^ conductances with the presence of the activator in S-ECS at the end and at the beginning of the experiment, respectively), calculated for each cell and plotted as a bar graph (mean ± SEM). Symbols show individual values, numbers in the bar indicate the number of cells. Statistical analysis was performed using unpaired Student’s t-tests (Riluzole vs. SKA-31) **(C)** and one-sample t-tests (against H_0_:μ_0_ = 1 hypothesis) **(C)**. ****p < 0.0001, n.s., not significant (*p* > 0.05). Extracellular pH was represented in all cases with the same colors: purple for 8.0, black for 7.4, green for 6.9, blue for 6.5 and red for 6.0.

To study the pH_e_-dependence of the effect of the KCa3.1 activators, Riluzole (5 µM) or SKA-31 (1 µM) were added to the extracellular solutions with pH_e_ ranging from 6.0 to 8.0. The intracellular pH in whole-cell patch-clamped PBLs was buffered at pH_i_ = 7.4, pH_i_ = 8.0 or pH_i_ = 6.5 using appropriate ICS solution (Table 1) and the intracellular free Ca^2+^ was maintained at 250 nM. This Ca^2+^ concentration allows robust activator effects to be measured (Jenkins et al., 2013). The raw current traces evoked by voltage ramps in Figure 4A; Supplementary Figure S3A show that the baseline KCa3.1 currents below the activation threshold of Kv1.3 are almost negligible using the 7.2-ICS-250 pipette filling solution containing 250 nM free Ca^2+^. Upon perfusing the recording chamber with the activators in S-ECS the slope of the current-voltage relationship increased drastically. Moreover, the slopes of the current traces recorded in the presence of the activators at various pH_e_ values are superimposable. The right-hand side panels show thea KCa3.1-specific K^+^ conductance values determined by the slope of the straight lines fitted to the current traces below the activation threshold of Kv1.3. The KCa3.1 conductance is instantaneously increased upon perfusion of either 1 µM SKA-31 (Figures 4B, D) or 5 µM Riluzole (Supplementary Figure S3B, D), regardless of the pH_e_ of the extracellular solution and the washout of the activators is completed in 2–4 episodes corresponding to 20–40 s. The reversible effect of the activators can be repeated over extended periods of time without significant loss in the KCa3.1 conductance (Figure 4E, left side). Qualitatively similar results were obtained when the 8.0-ICS-250 pipette filling solution was used (Figures 4C, D and Supplementary Figure S3C, D): the KCa3.1-specific K^+^ conductance increased remarkably in the presence of both Riluzole and SKA-31, the magnitude of the conductance was similar in all pH_e_ solutions and the reversible effect of the activators could be repeated over extended periods of time (Figure 4E, right side). The quantitative analysis in Figures 4F, G supported the above-mentioned observations. To compare the effect of the activators we first calculated the “fold increase in conductance” (see e.g., Figure 3B) caused by the activator at a given pH_i_ and pH_e_ combination and normalized it to the “fold increase in conductance” measured with the activators in S-ECS at pH_e_ = 7.4. The “fold increase normalized to 7.4” values obtained this way scatter around 1 for both Riluzole (Figure 4F) and SKA-31 (Figure 4G), regardless of the pH_i_ (7.2 or 8.0) or the pH_e_ (8.0, 6.9, 6.5 or 6.0). In summary, these data indicate that the potency of Riluzole and SKA-31 in activating the KCa3.1 current is independent of the extracellular pH, measured either using 7.2-ICS-250 or 8.0-ICS-250 solutions, i.e., when the pH_i_ was 7.2 or 8.0.

The response of the KCa3.1 current to Riluzole and SKA-31 is completely different from the above when the pipette-filling solution was 6.5-ICS-250, i.e., the pH_i_ was 6.5. Figures 5A, B show representative K^+^ conductance values recorded upon activation of the KCa3.1 current by Riluzole and SKA-31, respectively. The potency of both Riluzole and SKA-31 become progressively lost during continuous application of either of the drugs. To characterize the loss of the potency of the modulators over time we used the G_K,end_/G_K,start_ ratio where G_K,end_ and G_K,start_ are the modulator-enhanced K^+^ conductance at the end of the experiment (≥800 s) and the modulator-enhanced K^+^ conductance at the beginning of the experiment, respectively. The loss of the conductance was similar when 5 µM Riluzole or 1 µM SKA-31 were administered repeatedly and the phenomenon was independent of the pH_e_, i.e., it progressed continuously at all pH_e_ values until saturating at ∼20% of the activator-evoked conductance at the beginning of the experiment (Figure 5C). The cells, however, did not respond uniformly to the activators: 2 out of 12 cells displayed constant activator potency over time.

### 3.5 Potency of the KCa3.1 activator SKA-31 as a function of intra- and extracellular pH in CHO cells

**FIGURE 6:**
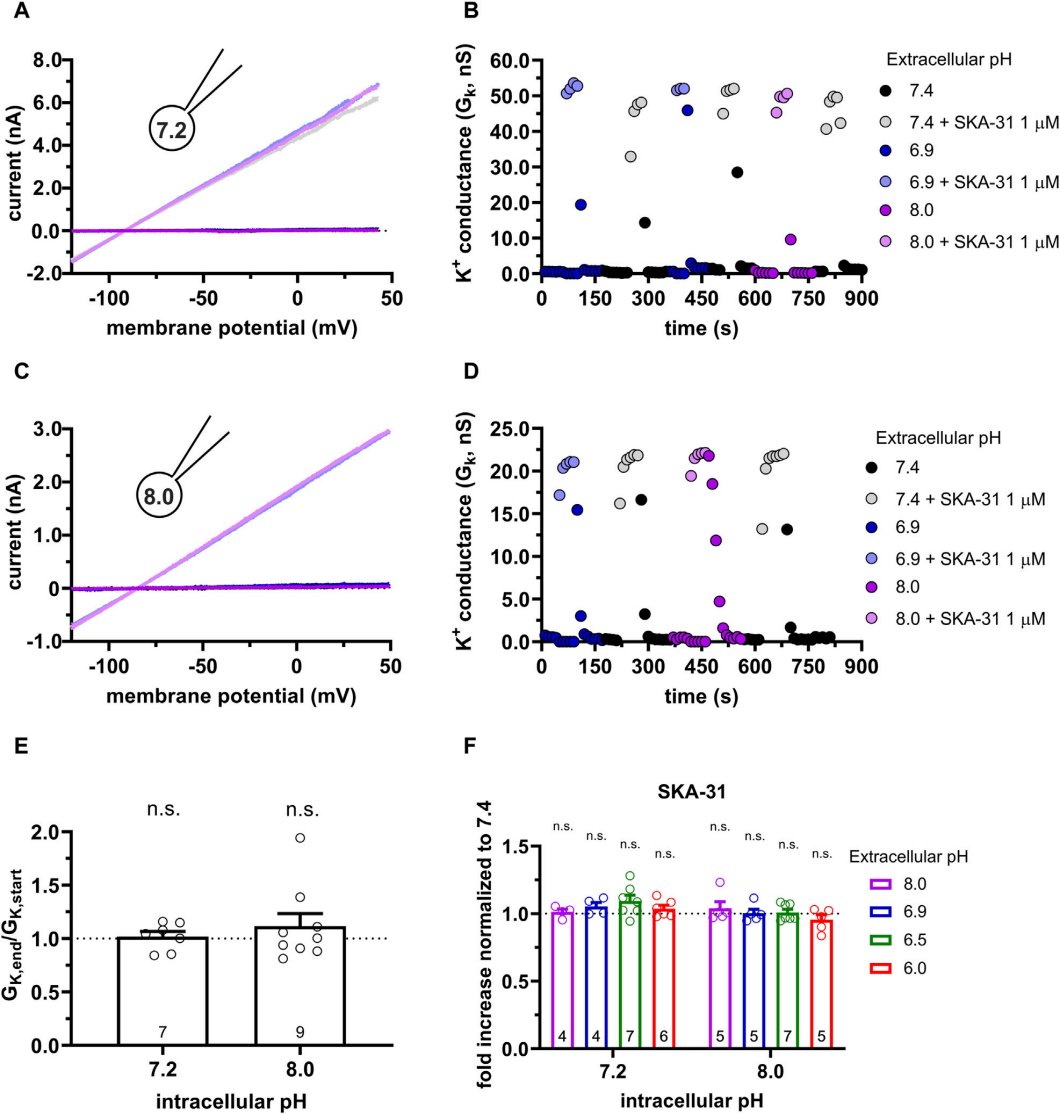
Effect of SKA-31 on the KCa3.1 currents expressed in CHO cells at different pH_e_ and pH_i_ combinations. **(A,C)** Representative current traces were evoked by 150-ms-long voltage ramps, ranging from −120 to +50 mV in whole-cell patch-clamped CHO cells. Voltage ramps were repeated every 10 s, the holding potential was −85 mV between pulses. The pipette filling solutions were 7.2-ICS-250 (pH_i_ = 7.2, panel A) or 8.0-ICS-250 (pH_i_ = 8.0, panel C). The cells were perfused with extracellular solutions having pH_e_ = 6.9 (6.9-ECS, blue), pH_e_ = 7.4 (S-ECS, black), and pH_e_ = 8.0 (8.0-ECS, purple). The corresponding lighter colors display traces obtained in the presence of the modulator. **(B,D)** KCa3.1-specific K^+^ conductance (G_K_) was determined by fitting straight lines to the traces panels A and C and plotted as a function of time. B: data from panel A, pH_i_ = 7.2. pH_e_ and 1 µM SKA-31 as indicated. D: data from panel B, pH_i_ = 8.0, pH_e_ and 1 µM SKA-31 as indicated. **(E)** Loss of the potency of SKA-31 was expressed as G_K,end_/G_K,start_ ratio (G_K,end_ and G_K,start_ are the averaged K^+^ conductances with the presence of the activator in S-ECS at the end and at the beginning of the experiment, respectively), calculated for each cell and plotted as a bar graph (mean ± SEM). Symbols show individual values, numbers in the bar indicate the number of cells. **(F)** The “fold increase normalized to 7.4” was calculated dividing the activator-induced “fold increase in conductance” (see e.g., Figure 3B) at a given pH_i_ and pH_e_ combination by the “fold increase in conductance” measured with the activators in S-ECS at pH_e_ = 7.4. Statistical analysis was performed using one-sample *t*-test **(E)** and one-way ANOVA (against H_0_:μ_0_ = 1 hypothesis) with multiple comparison (Bonferroni) **(F)**. n.s., not significant (*p* > 0.05). Extracellular pH was represented in all cases with the same colors: purple for 8.0, black for 7.4, green for 6.9, blue for 6.5 and red for 6.0.

We have demonstrated above that the characteristics of the potentiation of the KCa3.1 current by Riluzole and SKA-31 are similarly under the same experimental conditions (i.e., pH_e_-pH_i_ combinations). Therefore, we restricted the subsequent experiments to the more potent and selective SKA-31 and repeated the same set of experiments in CHO cells expressing KCa3.1 as in PBLs (see Figures 4, 5). Figure 6A shows whole-cell currents recorded in a CHO cell using 7.2-ICS-250 pipette filling solution. The current in the absence of SKA-31 (dark-toned colors) increases robustly upon perfusing the cells with solutions of different pH_e_ and supplemented with 1 µM SKA-31 (lighter-toned colors). The currents recorded in various pH_e_ values are superimposable, i.e., the potency of SKA-31 was the same regardless of the pH_e_. This is demonstrated more clearly in Figure 6B where the time-course of the experiment is displayed. The K^+^ conductance, determined from the slope of the current-voltage relationships, increases rapidly upon starting the perfusion with SKA-31-containing solutions. The effect of SKA-31 is quickly reversible upon perfusing the cell with activator-free solutions. The wash-in and wash-out of SKA-31could be repeated over an extended period without loss in the K^+^ conductance. Moreover, the K^+^ conductances measured in the presence of 1 µM SKA-31 were similar regardless of the pH_e_. The properties of the potentiation of the KCa3.1 current by SKA-31 were qualitatively the same when the activator was applied to a cell patch-clamped using 8-ICS-250 pipette filling solution (Figures 6C, D). The bar graph in Figure 6E addresses if the K^+^ conductance could be increased by SKA-31 over an extended period without decline in the potency of the activator. The data shown in Figure 6E were obtained by calculating G_K,end_/G_K,start_ ratio induced by 1 µM SKA-31 in S-ECS as described for Figure 5C. This ratio is ∼1 in Figure 6E which indicates that the potency of the activators remains constant throughout the experiment for both pH_i_ = 7.2 and pH_i_ = 8.0 pipette filling solutions. The observation that the effect of SKA-31 is independent of the pH_e_ of the recording solution is demonstrated quantitatively in Figure 6F. The “fold increase normalized to 7.4” variable (see above, description of Figure 4) values scatter around 1, like what was observed for PBLs (see Figure 4G). There was no statistical difference between this variable measured at various pH_e_ values when the pH_i_ of the pipette filling solution was either 7.2 or 8.0 (Figure 6F).

**FIGURE 7:**
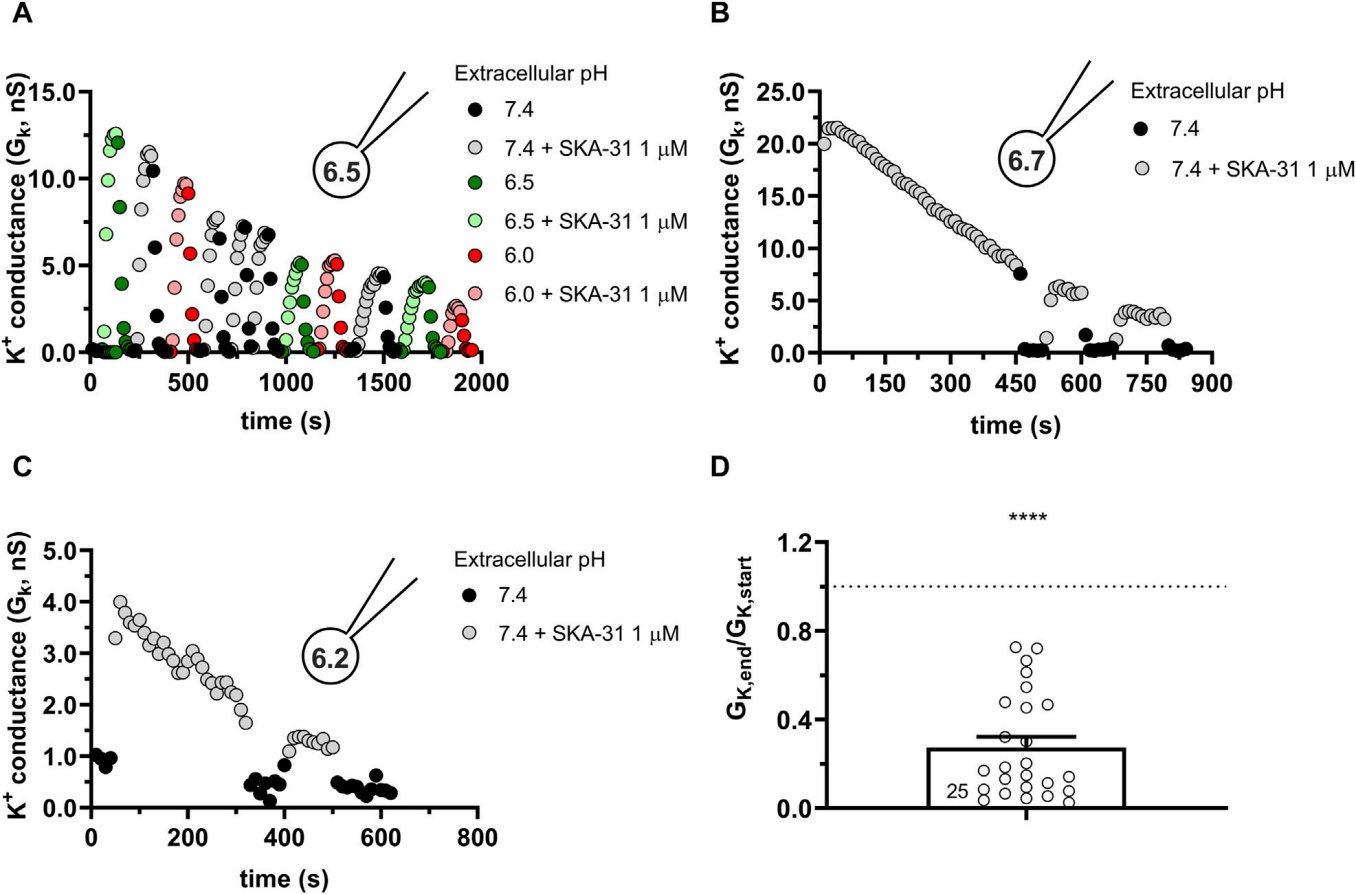
Progressive loss of the potency of SKA-31 in activating KCa3.1 currents CHO at intracellular acidic conditions. **(A)** Representative time course of the effect of SKA-31 when the intracellular solution was kept at pH_i_ = 6.5 (6.5-ICS-250) and the cell was perfused with extracellular solutions having different pH_e_ values (ranging from 6.0 to 7.4) and supplemented with 1 μM SKA-31, as indicated. KCa3.1 current traces were evoked by 150-ms-long voltage ramps, ranging from −120 to +50 mV in whole-cell patch-clamped CHO cells transfected with KCa3.1 channels. **(B,C)** Representative time course of the effect of SKA-31 when the intracellular solution was kept at pH_i_ = 6.7 **(B)** or pH_i_ = 6.2 **(C)** and the cell was perfused with S-ECS (pH_e_ = 7.4) and supplemented with 1 μM SKA-31, as indicated. Voltage protocol and other conditions as in panel A. **(D)** Loss of the potency of SKA-31 was expressed as G_K,end_/G_K,start_ ratio (G_K,end_ and G_K,start_ are the averaged K^+^ conductances with the presence of the activator in S-ECS at the end and at the beginning of the experiment, respectively), calculated for each cell and plotted as a bar graph (mean ± SEM). Symbols show individual values, numbers in the bar indicate the number of cells. Statistical analysis was performed using one-sample *t*-test (against H_0_:μ_0_ = 1 hypothesis) **(D)**. ****p < 0.0001. Extracellular pH was represented in all cases with the same colors: black for 7.4, green for 6.9, blue for 6.5 and red for 6.0. The corresponding lighter colors display traces obtained in the presence of the modulator.

Similar to the results obtained in PBLs the KCa3.1 conductance current progressively decreased when SKA-31 was repeatedly administered to the same cell at pH_i_ = 6.5 (6.5-ICS-250) (Figure 7). Figure 7A shows that the progressive loss of the SKA-31-induced conductance continued at an apparently similar rate regardless of the pH_e_ of the extracellular solution. Moreover, the K^+^ conductance could not be recovered upon re-application of SKA-31 in the control S-ECS solution with pH_e_ = 7.4. This loss-of-potency phenotype was also observed when the extracellular solution was kept constant, i.e., S-ECS, and pH_i_ was slightly more alkaline (pH_i_ = 6.7, Figure 7B) or more acidic (pH_i_ = 6.2 Figure 7C). Similar to PBLs, the cells did not respond uniformly to SKA-31, 5 out of 30 cells displayed relatively constant K^+^ currents upon repeated administration of the activator. The frequency of the cells showing stable K^+^ conductances vs. decline upon repeated SKA-31 application was statistically the same in PBLs and CHO cells (*p* > 0.80, Chi-square test with Yates’ correction). The loss of the K^+^ conductance over the time-course of the experiment was characterized quantitatively using the G_K,end_/G_K,start_ ratio as described for PBLs (see details in Figure 4E). The bar chart in Figure 7D shows that the cells showing loss-of-potency phenotype upon repeated SKA-31 application displayed a large scatter in G_K,end_/G_K,start_ ratio with a median of 0.17.

**FIGURE 8:**
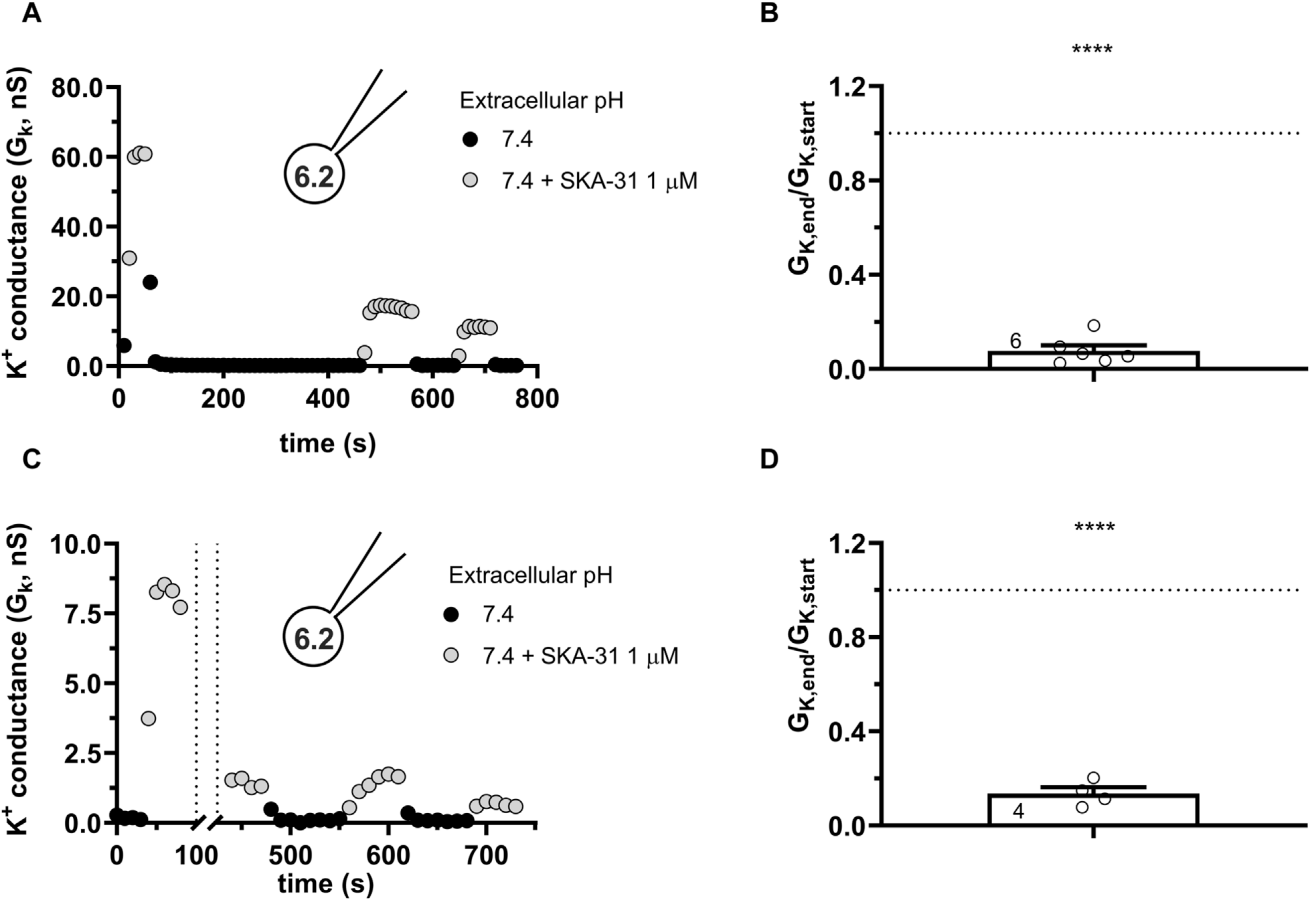
Progressive loss of the potency of SKA-31 in activating KCa3.1 currents during special SKA-31 administration protocols. KCa3.1 current traces were evoked by 150-ms-long voltage ramps, ranging from −120 to +50 mV in whole-cell patch-clamped CHO cells transfected with KCa3.1 channels. Voltage ramps were repeated every 10 s, the holding potential was −85 mV between pulses. The pipette filing solution was based on the 6.5-ICS-250 solution except that the pH_i_ was titrated to 6.2, the extracellular solution was S-ECS with or without 1 µM SKA-31 as indicated. **(A)** Representative time course of the effect of SKA-31 when the application of SKA-31 was interrupted for 400 s. **(B)** Loss of the potency of SKA-31 was expressed as G_K,end_/G_K,start_ ratio (G_K,end_ and G_K,start_ are the averaged K^+^ conductances with the presence of the activator in S-ECS at the end and at the beginning of the experiment, respectively), calculated for each cell and plotted as a bar graph (mean ± SEM). Symbols show individual values, numbers in the bar indicate the number of cells. Panel B refers to panel A. **(C)** Representative time course of the effect of SKA-31 when the application of the voltage ramps was interrupted for 360 s in the continuous presence of 1 µM SKA-31. **(D)** Loss of the potency of SKA-31 relative to panel C. Except the pulse protocol, all other details are the same as in Panel B. Panel D refers to panel C. Statistical analysis was performed using one-sample t-test (against H_0_:μ_0_ = 1 hypothesis) **(B,D)**. ****p < 0.0001.

To get more insight into the nature of loss-of-potency phenotype we studied the response of the currents to SKA-31 at a constant pH_e_ of 7.4 (S-ECS) and pH_i_ of 6.2 and varied the pattern of SKA-31 application. The first special protocol started with SKA-31 administration at the beginning of the experiment followed by a washout (Figure 8A). Thereafter the cell was repeatedly depolarized using voltage ramps every 10 s for 400 s in the absence of SKA-31. SKA-31 was reapplied after this period but failed to potentiate the current to the same extent as in its first application to this cell. The quantitative analysis of the drop in the potentiation of the K^+^ conductance is in Figure 8B. The G_K,end_/G_K,start_ ratio (see above) is significantly smaller than 1 thereby indicating the decline in the potentiation of the K^+^ conductance by the end of the experiments. Thus, the loss-of-potency phenotype cannot be prevented by inserting a drug-free period into the protocol where voltage ramps are repeatedly applied. In the next special protocol (Figure 8C) the KCa3.1 conductance was first potentiated by SKA-31 application. Thereafter, while the SKA-31-containng solution was perfused constantly on the cell, the delivery of the voltage ramps was suspended for 360 s and the cell was kept at the holding potential of −85 mV during this period. The repeatedly applied voltage ramps restarted after the gap. As Figure 8C shows that the SKA-31-induced KCa3.1 conductance was much smaller after the gap in the recording than at the beginning of the experiment (Figure 8D). The G_K,end_/G_K,start_ ratio (see above) is significantly smaller than 1 thereby confirming the loss-of-potency phenotype by the end of the experiments. Thus, the decline in the potency of SKA-31 can neither be prevented by inserting a voltage-ramp-free period into the protocol, nor by inserting a drug-free period into the protocol, indicating that the loss-of-potency phenotype must be attributed to the acidic pH_i_.

### 3.6 The activation of KCa2.2 by SKA-31 is also sensitive to the intracellular pH of the recording solution

**FIGURE 9:**
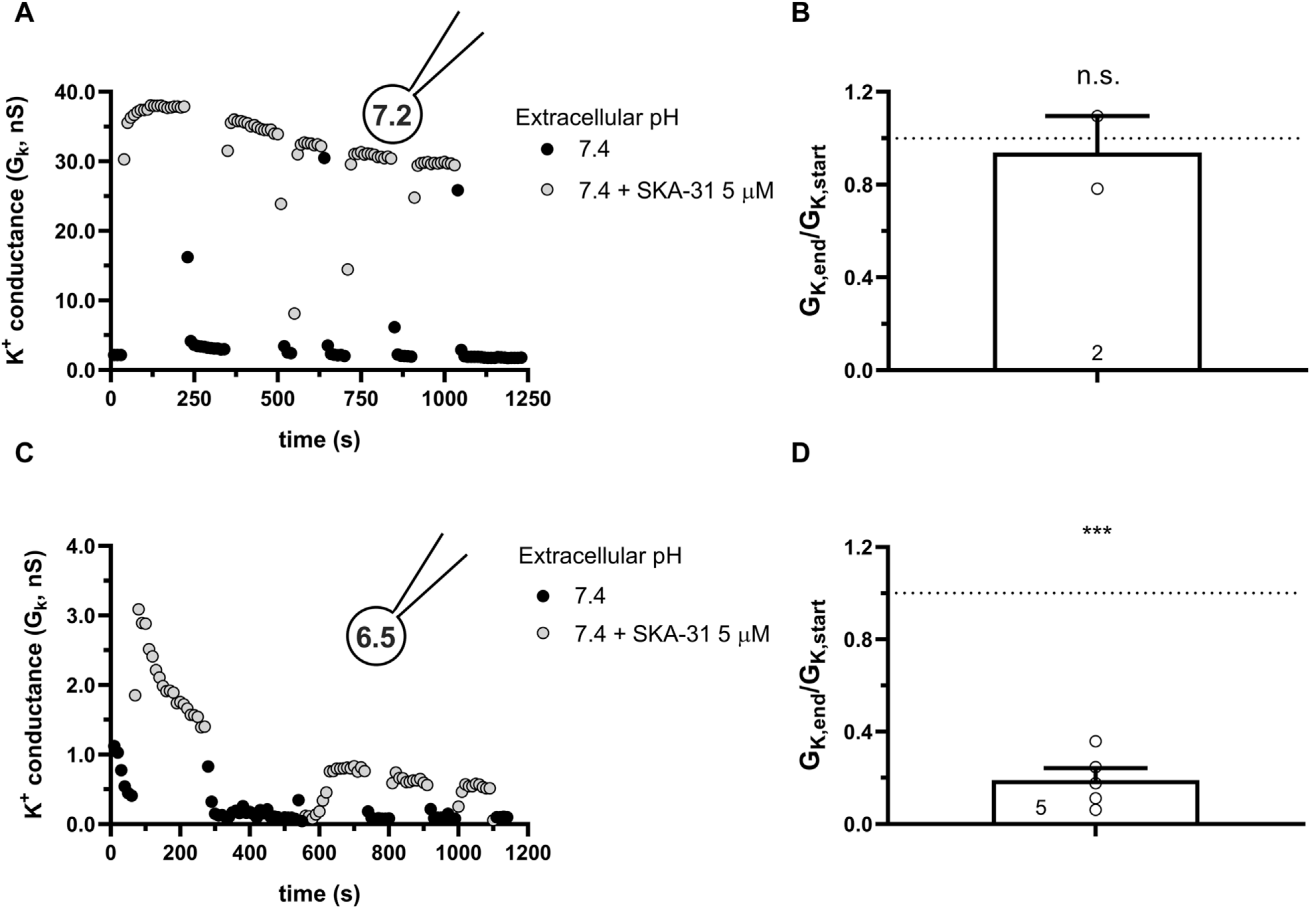
Effect of SKA-31 on currents generated in CHO cells transfected with hKCa2.2 and GFP at different intracellular pH. **(A,C)** KCa3.1 current traces were evoked by 150-ms-long voltage ramps, ranging from −120 to +50 mV in whole-cell patch-clamped CHO cells transfected with KCa3.1 channels. Voltage ramps were repeated every 10 s, the holding potential was −85 mV between pulses. The pipette filing solution was either pH_i_ = 7.2 (7.2-ICS-250) **(A)** or pH_i_ = 6.5 (6.5-ICS-250) **(C)**. The extracellular solution was S-ECS with or without 5 µM SKA-31 as indicated. **(B,D)** Loss of the potency of SKA-31 was expressed as G_K,end_/G_K,start_ ratio (G_K,end_ and G_K,start_ are the averaged K^+^ conductances with the presence of the activator in S-ECS at the end and at the beginning of the experiment, respectively), calculated for each cell and plotted as a bar graph (mean ± SEM). Symbols show individual values, numbers in the bar indicate the number of cells. B refers to A and D refers to C. Statistical analysis was performed using one-sample *t*-test (against H_0_:μ_0_ = 1 hypothesis) **(B,D)**. ***p < 0.001, n.s., not significant (*p* > 0.05).

KCa3.1 and the members of the KCa2.x family share several structural features: they are made up of four alpha subunits, each containing six transmembrane domains and a Calmodulin-binding domain in the C-terminal portion. KCa2.x channels are activated by intracellular Ca^2+^ and the same modulators as KCa3.1 (Guéguinou et al., 2014), albeit KCa2.x channels are ∼10 times less sensitive to molecules like Riluzole and SKA-31 than KCa3.1. We chose KCa2.2 as the representative channel of this family and assessed the ability of SKA-31, at a higher concentration of 5 μM, to activate the current at neutral and acidic intracellular pH conditions. At pH_i_ of 7.2 (Figure 9A) the robust activation of the KCa2.2 by SKA-31 can be repeatedly evoked with negligible loss in the modulator’s efficacy over time. On the contrary, when the intracellular solution was set at pH_i_ = 6.5 a rapid and irreversible loss of the potency of SKA-31 to activate KCa2.2 was observed (Figure 9C), similar to the findings for KCa3.1. The quantitative analysis of the data in Figures 9B, D show very similar results to the ones obtained in CHO cells transfected with hKCa3.1, i.e., there is a significant loss in the potency of SKA-31 to activate KCa2.2 at acidic intracellular pH (cfr. Figures 6E, 7D).

### 3.7 The mutations H192A in hKCa3.1 and T79D in Calmodulin do not interfere with the loss of potency of SKA-31 due to intracellular acidity

**FIGURE 10:**
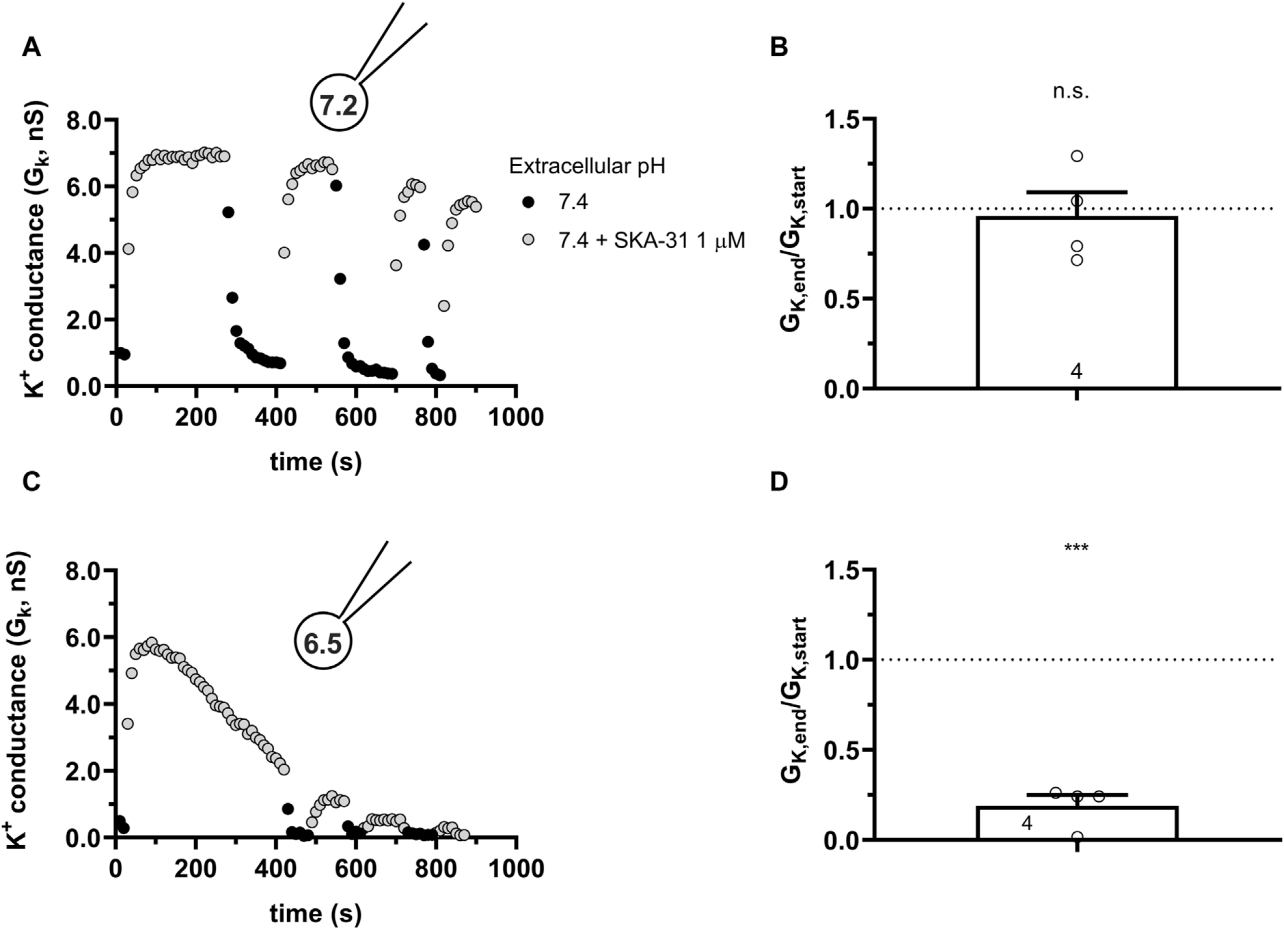
Effect of SKA-31 on currents generated in CHO cells transfected with the mutated H192A-hKCa3.1 and GFP at different intracellular pH. **(A,C)** KCa3.1 current traces were evoked by 150-ms-long voltage ramps, ranging from −120 to +50 mV in whole-cell patch-clamped CHO cells transfected with KCa3.1 channels. Voltage ramps were repeated every 10 s, the holding potential was −85 mV between pulses. The pipette filing solution was either pH_i_ = 7.2 (7.2-ICS-250) **(A)** or pH_i_ = 6.5 (6.5-ICS-250) **(C)**. The extracellular solution was S-ECS with or without 1 µM SKA-31 as indicated. **(B,D)** Loss of the potency of SKA-31 was expressed as G_K,end_/G_K,start_ ratio (G_K,end_ and G_K,start_ are the averaged K^+^ conductances with the presence of the activator in S-ECS at the end and at the beginning of the experiment, respectively), calculated for each cell and plotted as a bar graph (mean ± SEM). Symbols show individual values, numbers in the bar indicate the number of cells. B refers to A and D refers to C. Statistical analysis was performed using one-sample *t*-test (against H_0_:μ_0_ = 1 hypothesis) **(B,D)**. ***p < 0.001, n.s., not significant (*p* > 0.05).

A recent cryo-EM derived structure of hKCa3.1 revealed a role of the S4-S5 linker in the formation of the functional and structural connection between Calmodulin and the C-terminal portion of the KCa channels (Lee and MacKinnon, 2018). Residue His192 is in this linker and faces the pocket where the activators are thought to exert their effect. Mutating this histidine to a non-charged alanin (H192A) caused the disruption of the interaction between BA6b9, a KCa3.1 blocker devised to be structurally similar to Riluzole/1-EBIO and KCa3.1 (Burg et al., 2022). Moreover, it is known that protonation of histidine residues in acidic environments can disrupt the ability of toxins to bind to ion channels, making the toxins less or non-functional (Aiyar et al., 1995). Considering the structural similarity between BA6b9 and the modulators used in this study and that they fit in overlapping binding pockets, we checked whether the H192A mutation would influence the activation of hKCa3.1 by SKA-31 in neutral and in acidic conditions. Figure 10A shows that SKA-31 activates the H192A-KCa3.1 current, in a reversible manner, similar to the wild type KCa3.1. The wash-in-wash-out cycles could be repeated several times when the intracellular solution was set to 7.2. The potency of SKA-31 in activating the current was relatively constant over extended periods of time (Figure 10B). However, at pH_i_ = 6.5 SKA-31 gradually lost its potency in activating the H192A-KCa3.1 current during repeated administration (Figure 10C, D). This suggests that the loss of SKA-31 potentiation of the current at acidic intracellular pH is oblivious to whether the protonated His or the neutral Ala is in position 192.

**FIGURE 11:**
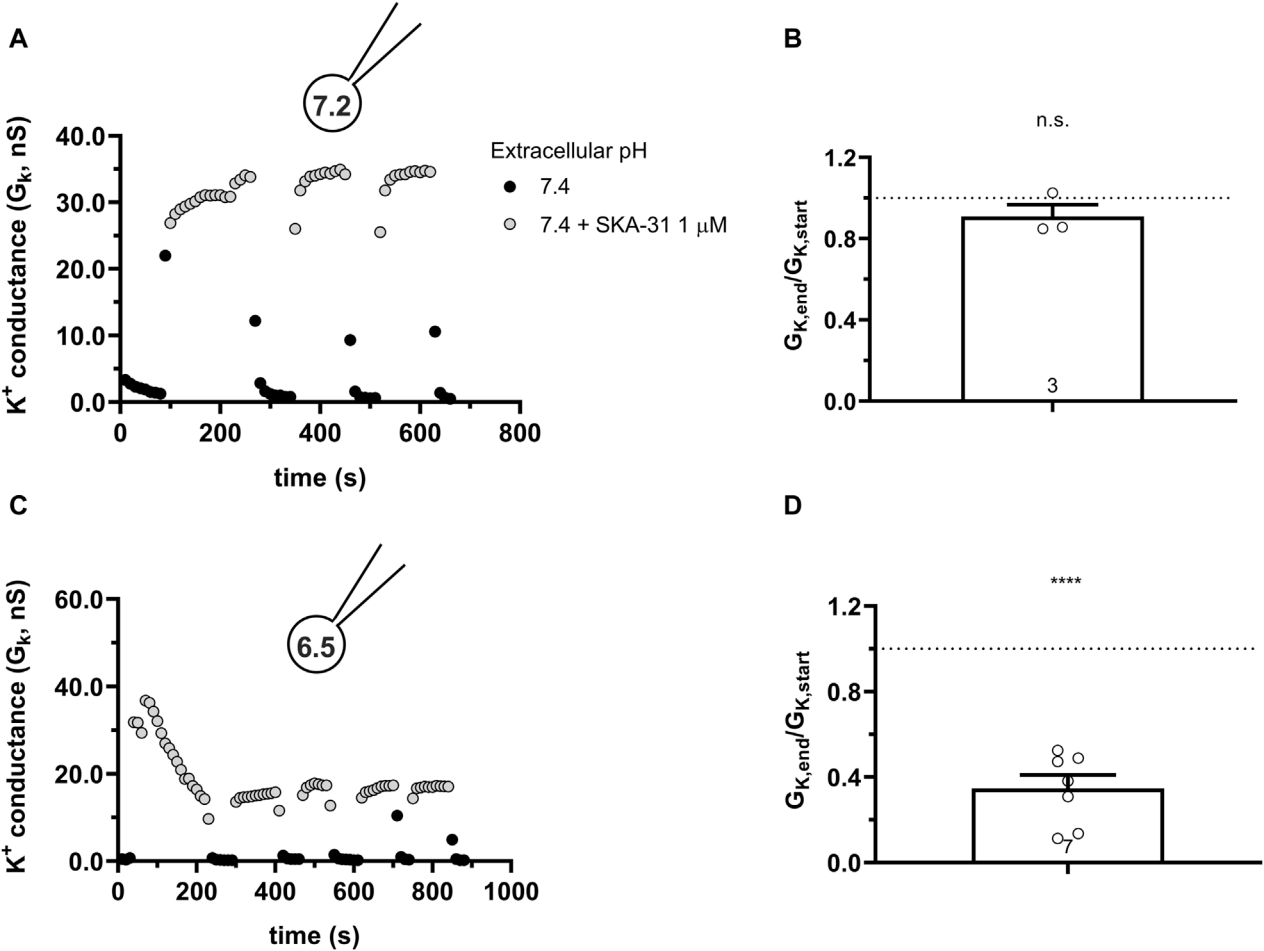
Effect of SKA-31 on currents generated in CHO cells transfected with turboGFP-hKCa3.1 and the mutated T79D-CaM at different intracellular pH. **(A,C)** KCa3.1 current traces were evoked by 150-ms-long voltage ramps, ranging from −120 to +50 mV in whole-cell patch-clamped CHO cells transfected with KCa3.1 channels. Voltage ramps were repeated every 10 s, the holding potential was −85 mV between pulses. The pipette filing solution was either pH_i_ = 7.2 (7.2-ICS-250) **(A)** or pH_i_ = 6.5 (6.5-ICS-250) **(C)**. **(B,D)** Loss of the potency of SKA-31 was expressed as G_K,end_/G_K,start_ ratio (G_K,end_ and G_K,start_ are the averaged K^+^ conductances with the presence of the activator in S-ECS at the end and at the beginning of the experiment, respectively), calculated for each cell and plotted as a bar graph (mean ± SEM). Symbols show individual values, numbers in the bar indicate the number of cells. B refers to A and D refers to C. Statistical analysis was performed using one-sample *t*-test (against H_0_:μ_0_ = 1 hypothesis) **(B,D)**. ****p < 0.0001, n.s., not significant (*p* > 0.05).

Calmodulin (CaM) is constitutively bound to KCa channels (Lee and MacKinnon, 2018) which, besides Ca^2+^ ions, also requires membrane-bound PIP_2_ as co-agonist (Burg et al., 2022).Thr79 in CaM is the target of the Casein Kinase-2 (CK2) (Bildl et al., 2004). When Thr79 is phosphorylated, it results in the loss of the sensitivity of the KCa2.2 channel to PIP_2_ (Zhang et al., 2014) and Ca^2+^ (Allen et al., 2007). The phosphorylation of Thr79 can be mimicked by the phosphomimetic mutation T79D, this mutation decreases the K^+^ current both in KCa2.2 (Zhang et al., 2014) and KCa3.1 (Burg et al., 2022). The disturbed network of activators and co-activators in the presence of T79D-CaM may be reflected in the modulation of KCa3.1 activation by SKA-31 at acidic pH_i_. To test this, we co-transfected hKCa3.1 and T79D-CaM into CHO cells and studied the potentiation of the whole-cell current at neutral and acidic pH_i_. Figures 11A, B show that using neutral pH_i_ condition the current could be activated by SKA-31 similar to what was obtained in cells transfected with hKCa3.1 only (see Figure 6). The activation cycles by SKA-31 resulted in consistently increased K^+^ conductance over extended time periods (Figure 11B). On the contrary, when the pH_i_ = 6.5 was used SKA-31 gradually lost its potency over time to activate G_K_ (Figures 11C, D). The average loss of the G_K_ by the end of the experiment (>800 s, Figure 11D) is slightly reduced as compared to when CHO cells were transfected with wild-type (Figure 7D) or H192A KCa3.1 constructs (Figure 10D), but the G_K,end_/G_K,start_ ratio was non significantly different among the three groups (One-way ANOVA, *p* > 0.05). Moreover, some cells displayed a very slow restoration of the potency of SKA-31 over time (Figure 11C). This phenomenon was not investigated any further due to inherent limitations of the whole-cell patch-clamp over extended durations beyond 15–20 min.

### 3.8 High intracellular Ca^2+^ concentration hinders the inhibitory effect of intracellular acidity

**FIGURE 12:**
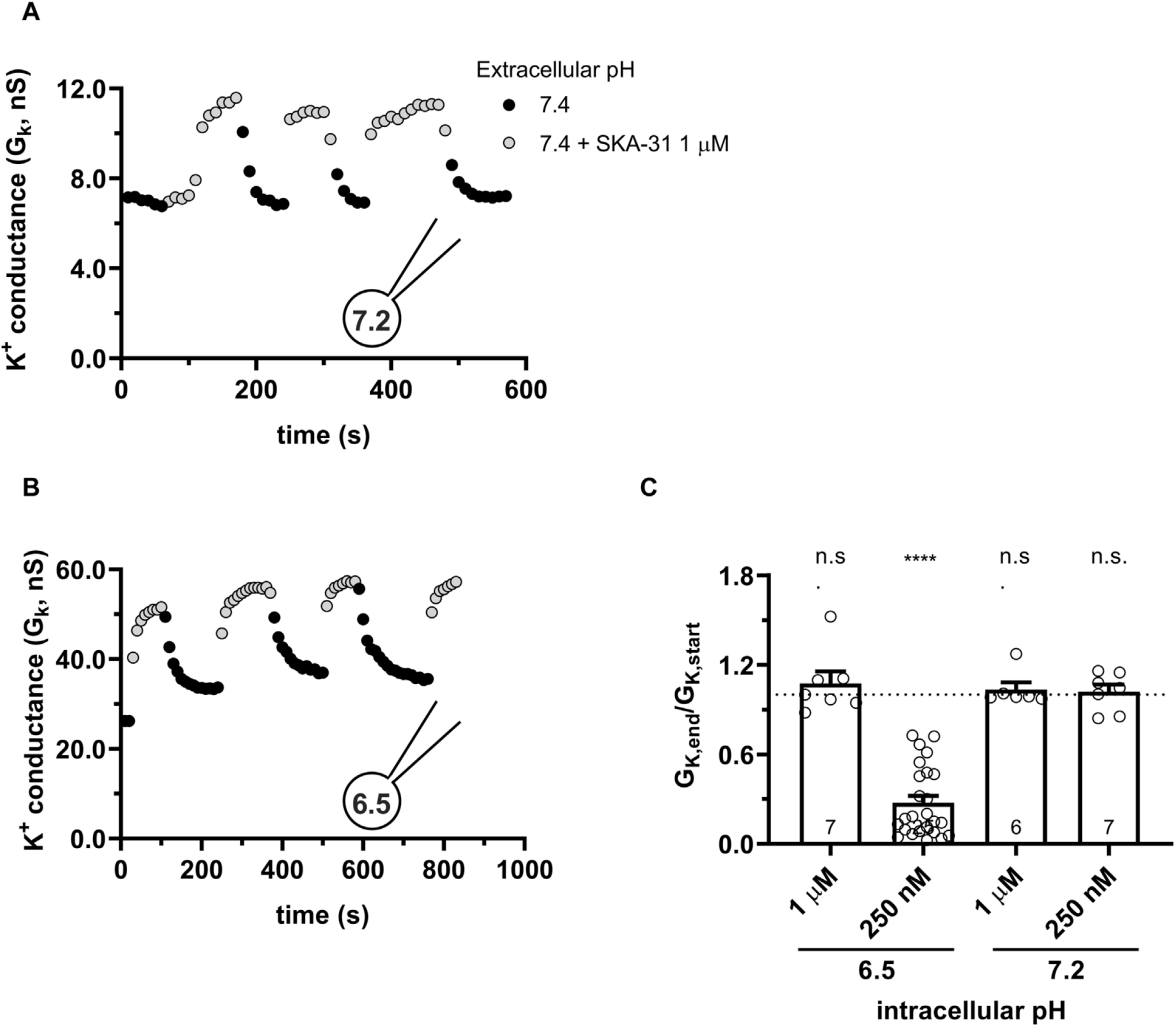
High intracellular Ca^2+^ (1 μM) sustains the functionality of SKA-31. **(A,B)** KCa3.1 current traces were evoked by 150-ms-long voltage ramps, ranging from −120 to +50 mV in whole-cell patch-clamped CHO cells transfected with KCa3.1 channels. Voltage ramps were repeated every 10 s, the holding potential was −85 mV between pulses. The pipette filing solution was at pH_i_ = 6.5 and 1 μM Ca^2+^ (6.5-ICS) **(A)** and at pH_i_ = 7.2 and 1 μM Ca^2+^ (S-ICS) **(B)**. The extracellular solution was S-ECS with or without 1 µM SKA-31 as indicated. **(C)** Loss of the potency of SKA-31 was expressed as G_K,end_/G_K,start_ ratio (G_K,end_ and G_K,start_ are the averaged K^+^ conductances with the presence of the activator in S-ECS at the end and at the beginning of the experiment, respectively), calculated for each cell and plotted as a bar graph (mean ± SEM). Symbols show individual values, numbers in the bar indicate the number of cells. Statistical analysis was performed using one-way ANOVA (against H_0_:μ_0_ = 1 hypothesis) with multiple comparison (Bonferroni) **(C)**. ****p < 0.0001. n.s., not significant (*p* > 0.05).

KCa3.1 is extremely sensitive to the intracellular concentration of Ca^2+^, presenting an EC_50_ ranging from 100 to 400 nM (Brown et al., 2019) and a characteristic sigmoid activation curve (Bailey et al., 2010). When SKA-31 was applied to hKCa3.1-expressing CHO and 1 μM Ca^2+^ concentration was used in the pipette at pH_i_ of 7.2 we found the expected: 1) an elevated base-line KCa3.1 conductance due to the higher intracellular Ca^2+^ (Figures 12A, B) and 2) a reduced potentiation of G_K_ by SKA-31 (∼2 fold vs. ∼50-fold at 250 nM cytosolic Ca^2+^ concentration, see Figure 3) due to near-saturation levels in the Ca^2+^ sensitivity of the channel. Interestingly, at 1 μM cytosolic Ca^2+^ concentration the potency of SKA-31 to upregulate KCa3.1 conductance remained constant even at acidic pH_i_ = 6.5 (Figure 12B). The G_K,end_/G_K,start_ parameter obtained at pH_i_ = 6.5 and 1 μM Ca^2+^ did not differ statistically from the data obtained at pH_i_ = 7.2 at either 1 µM or 250 nM cytosolic Ca^2+^ concentration (Figure 12C). This means that at saturating concentration of intracellular Ca^2+^, the potency of SKA-31 in activating KCa3.1 remains constant regardless of the pH_i_.

## 4 Discussion

To our knowledge, our paper is the first comprehensive study that analyzes how extra- and intracellular pH influences the magnitude of the hKCa3.1 current and its potentiation by the positive modulators of the channel SKA-31 and Riluzole. We showed that the hKCa3.1 current expressed endogenously in human peripheral blood lymphocytes or expressed heterologously in CHO cells shows very subtle sensitivity to the pH_i_ ranging from 6.5 to 8.0 and pH_e_ ranging from 6.0 to 8.0. The very potent activators of KCa3.1, Riluzole, and SKA-31 induce robust KCa3.1 currents at normal (pH_i_ = 7.2) and alkaline (pH_i_ = 8.0) intracellular pH for both endogenously and heterologously expressed channels. On the other hand, the potency of SKA-31 in activating the KCa3.1 current declines over time when the intracellular pH is acidic (pH_i_ < 6.5). The loss of the potency of SKA-31 was not specific for KCa3.1, the potentiation of the current also declined over time when KCa2.2 was studied at pH_i_ = 6.5. The loss of the SKA-31 potency at acidic pH_i_ was also shown for a KCa3.1 mutant where a titratable His was mutated to Ala (H192A) in the binding pocket for the activators. Similarly, transfection of CHO with T79D, a Calmodulin mutant that confers reduced Ca^2+^ sensitivity to KCa3.1, did not prevent the loss-of-potency phenotype when SKA-31 was applied at acidic pH_i_. However, increasing the cytosolic Ca^2+^ concentration to 1 µM eliminated the loss-of-potency phenotype of SKA-31 activation at acidic pH_i_.

The dependence of the K^+^ conductance on the extracellular pH may originate from at least two sources. One is related to the screening of the surface charges when the H^+^ concentration is increased, i.e., pH_e_ is lowered. Screening of the surface charges will affect the operation of the voltage sensor domain (VSD) of voltage-gated channels, as was demonstrated for Shaker (Broomand et al., 2007) and Kv1.3 (Deutsch and Lee, 1989; Teisseyre and Mozrzymas, 2007), among others. KCa3.1 lacks the charged S4 helix in the VSD and is not a voltage-gated ion channel, therefore, the lack of the effect of pH_e_ on the K^+^ conductance in KCa3.1 is not surprising. pH_e_ can also regulate ion channels by interacting specifically with amino acid residues exposed to the extracellular solution. This was demonstrated e.g., for the Na^+^-permeable ASIC ion channels (acid-sensing ion channels) (Gonzales et al., 2009; Cheng et al., 2018). Interestingly, acidic extracellular pH influences drastically the conductance, inactivation kinetics, and pharmacology of Kv1.3 due to the presence of a titratable His residue in the entrance of the ion-conducting pore in each subunit of the tetrameric channel (Deutsch and Lee, 1989; Somodi et al., 2004; Somodi et al., 2008). The human KCa3.1 contains a valine at an equivalent position (V257). The titratable amino acid residues near the selectivity filter are H236 near the pore helix and D239 in the pore helix, but even if these residues are protonated at acidic pH_e_ it does not influence drastically the K^+^ conductance of KCa3.1 channels. Acidic pH_e_ significantly lowers the K^+^ currents through hKCa3.1, but the current loss never exceeded 15%–20% as compared to pH_e_ = 7.4. Moreover, this effect can be mostly observed at extracellular pH 6.0, which is very low and unlikely in either a physiological or pathological context.

Many voltage-gated K^+^ channels are also affected by pH_i_. For example, the whole-cell Kv1.3 current in PBLs was enhanced by alkaline and inhibited by acidic pH_i_ (Deutsch and Lee, 1989). The pH_i_-dependence of the conductance was attributed to a change in the number of channels that open and the change in the single-channel conductance. The current reduction in Shaker-IR K^+^ channels at acidic pH_i_ is caused by a reversible block of the channels by protons (Starkus et al., 2003). The proton block of Shaker IR was accompanied by a significant reduction of the single-channel current and specific interaction of protons with amino acid side chains in the internal vestibule of the channels was proposed, but the side chains mediating this effect were not identified. Although the general pore architecture, with cytoplasmic activation gate at Val282, and the selectivity filter are similar in Kv channels and KCa3.1, the dependence of the KCa3.1 conductance on pH_i_ is virtually absent, as shown in our study.

Based on the insensitivity of the KCa3.1 current to the pH_i_-pH_e_ combinations we conclude that the gating machinery of KCa3.1 (Lee and MacKinnon, 2018) and the network of co-activators (Ca^2+^, CaM, and PiP_2_) is not affected by pH_i_ and pH_e_ relevant to the physiological and pathophysiological conditions. Our conclusion apparently contradicts previous studies where the pH sensitivity of the shape (Pandey et al., 2014), the Ca^2+^ binding capacity (Valeyev et al., 2008), and the Ca^2+^ affinity (Iida and Potter, 1986) of CaM were reported. These latter results were obtained either using isolated CaM in solution or by mathematical modeling, which may explain the difference between these studies and ours.

Voltage- and Ca^2+^-activated channels can be inhibited by small molecules and/or peptide blockers. A remarkable pharmacological feature of KCa3.1 is that a group of small molecules based on the structure of EBIO-1 (Brown et al., 2019) act as activators of the channel. These activators can be used experimentally to boost channel function and consequently modulate physiological and pathophysiological responses cells (see below). The mechanism of action of the activators is that they shift the calcium-activation curve in a concentration-dependent manner towards lower intracellular Ca^2+^ concentrations, thereby increasing the apparent Ca^2+^ affinity, but are unable to activate the channels in the absence of intracellular Ca^2+^. In that respect they are positive-gating modulators, however, they also exert a super-agonist effect whereby they activate the current even at a saturating concentration of cytosolic Ca^2+^, as it is also shown for activation of KCa3.1 by SKA-31 at 1 µM Ca^2+^ concentration. Based on the structures of Riluzole and SKA-31 and their predicted pKa values (2.96 and 3.5) the change in the protonation of the molecules in the pH range between 6.0 and 8.0 is negligible. In line with this, SKA-31 and Riluzole potentiated the KCa3.1 current for all pH_e_-pH_i_ combinations as long as the pH_i_ remained neutral or basic.

On the contrary, when the pH_i_ was acidic, both SKA-31 and Riluzole lost their potency in activating KCa3.1 over the several hundred seconds time-course of our experiments. A trivial explanation for the loss-of-potency phenotype could be that the exposure of SKA-31 and Riluzole to acidic pH_i_ may cause a structural change in the activator molecule that is irreversible and develops over the extended time course of the experiments. Based on our data this is unlikely, Riluzole and SKA-31 maintained their potency when they were dissolved in pH_e_ = 6.0 extracellular solution. All extracellular solutions, including the pH_e_ = 6.0 + SKA/Riluzole, were used all day without losing the potency of the activators. Moreover, immediately upon the application of SKA-31 or Riluzole the KCa3.1 current was potentiated even when the pH_i_ was acidic. This means that access of SKA-31 and Riluzole to the modulatory site, including membrane permeation, is not compromised at either pH_i_-pH_e_ combination. Moreover, once we exposed the intracellular environment to acidic pH_i_ the loss of the SKA-31-mediated current activation progressed when we interrupted SKA-31 application or interrupted the current recordings for several hundred seconds (Figure 8). The only manipulation that prevented the loss-of-potency phenotype was the increase in the cytosolic Ca^2+^ concentration to 1 μM (Figure 12).

Based on the above the loss-of-potency phenotype may associated with the altered Ca^2+^-dependence of KCa3.1 gating in the presence of the activators and/or by a pH_i_-dependent alteration of the binding activator binding site. This motivated us to study if key residues in the vicinity of the putative binding pocket for KCa3.1 activators influence the loss-of-potency phenotype at acidic pH_i_. The binding site for the positive gating modulators was proposed based on the cryo-EM structure of KCa3.1/CaM complex (Lee and MacKinnon, 2018) to the interface between the S_4-5_A helix of KCa3.1 and the N-lobe of CaM. Later the binding site for the SKA-31 analogue SKA-111 was localized into this pocket using Rosetta modelling (Shim et al., 2019). This binding pocket is in the immediate vicinity of the interacting surface with the head group of PIP_2_ and to the site where BA6b9, a blocker structurally similar to Riluzole/1-EBIO binds (Burg et al., 2022). The BA6b9 binding site involves H192 in the S_4-5_B helix of KCa3.1, which may be protonated at acidic pH_i_ and thus, influence the interactions among amino acid side chains in this critical region leading to the loss of the potency of the KCa3.1 activators. However, the following lines of evidence argue against this scenario: i) the H192A mutant of KCa3.1 shows the loss-of-potency phenotype at acidic pH_i_ although the mutant channel cannot be protonated at position 192; ii) the KCa2.2 channel, that contains a Threonin (T) at equivalent position also shows the loss-of-potency phenotype at acidic pH_i_. Moreover, the loss-of-potency phenotype persisted in the presence of the T79D mutant of CaM. T79D mimics the phosphorylation of T79 which leads to a lower sensitivity of KCa3.1 activation by PIP_2_ and Ca^2+^ (Burg et al., 2022). This phenomenon may be the consequence of structural changes in the strategically designed S4-S5 linker region and its vicinity in T79D. Nevertheless, the T79D mutation of CaM did not alter the behavior of the activators at acidic pH_i_.

For the loss of the activator-induced KCa3.1 conductance at acidic pH_i_, it may also be envisioned that the combination of acidic pH_i_, low (250 nM) Ca^2+^, and the presence of the activators leads to a decreased availability of the channels to open. This warrants further experiments which may include the analysis of His358 phosphorylation KCa3.1 at various pH_i_ values and its consequences on CaM-dependent activation of the channels (Srivastava et al., 2006; 2016; Ji et al., 2018; Zechel et al., 2019).

Although our efforts in isolating the molecular mechanism for the loss of the potency of KCa3.1 activators in acidic pH_i_ are inconclusive at this moment, the phenomenon is interesting and may have significant consequences regarding the use of KCa3.1 activators in experimental settings. The pharmacological activation of KCa3.1 using positive modulators has been proposed as a novel way to boost the suppressed immune system in its fight against cancer (Chandy and Norton, 2016; Brown et al., 2017) [reviewed recently in (Chirra et al., 2022)]. This seems to be important to overcome the immunosuppressive TME caused by high extracellular K^+^ (Eil et al., 2016), adenosine concentration (Chiarella et al., 2021), and severe acidity (Huber et al., 2017). For example, activation of KCa3.1 channels by 1-EBIO restored the ability of cancer-derived CD8^+^ T cells to chemotax in the presence of adenosine (Chimote et al., 2018) and rescued T cell function *in vitro* in high extracellular [K^+^] that is characteristic to the TME (Eil et al., 2016). Considering that in the acidic TME, the cytosolic pH is also acidic (Navarro et al., 2022) the benefits of KCa3.1 positive modulators can be compromised by the loss-of-potency phenotype at acidic pH_i_ described in this study. On the other hand, several cancer types such as glioblastoma (Brown et al., 2017), pancreatic ductal adenocarcinoma (Soret et al., 2023), prostate cancer (Ohya et al., 2009), non-small cell lung cancer (Bulk et al., 2015) and breast cancer (Gross et al., 2022) overexpress KCa3.1. In these cases, the use of an activator would be potentially counterproductive and the loss of the potency of the activators in the acidic TME may be beneficial. So the overall outcome of the acidic pH_i_-induced loss of the potency of KCa3.1 activators must be evaluated for both the immune system and the cancer cells.

## Data Availability

The raw data supporting the conclusion of this article will be made available by the authors, without undue reservation.
