# Supplementary material for: Intracellular acidity impedes KCa3.1 activation by Riluzole and SKA-31
Source: Front Pharmacol. 2024 Apr 4;15:1380655. doi: 10.3389/fphar.2024.1380655 (PMC11024243; doi:10.3389/fphar.2024.1380655)
Supplement: Supplementary file 1 [file DataSheet1.PDF]

Supplementary Figures

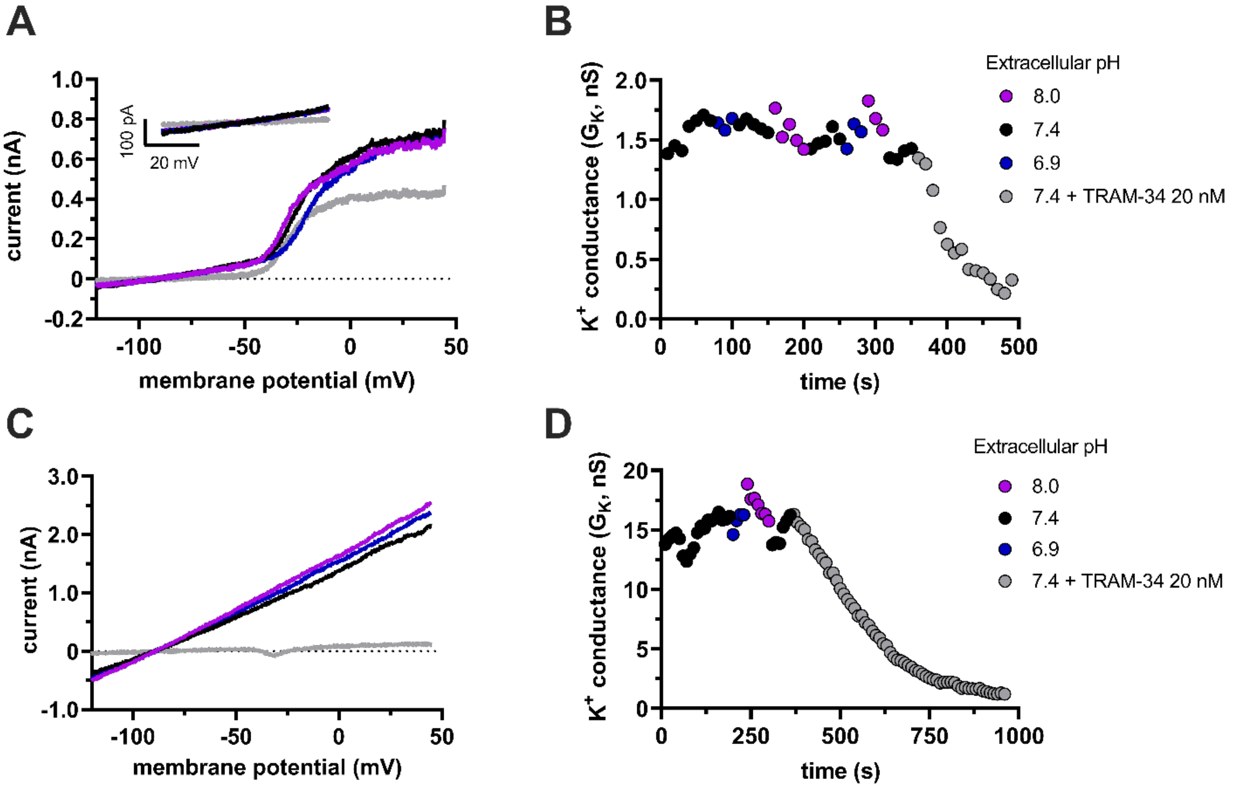

Supplementary Figure 1. TRAM-34 sensitivity of the whole-cell current in PBLs and in CHO cells. (**A**, **C**) Representative current traces were evoked by 150-ms-long voltage ramps, ranging from −120 to +50 mV in a whole-cell patch-clamped human peripheral T cell (A) or CHO cell (B). Voltage ramps were repeated every 10 seconds, the holding potential was −85 mV between pulses. The pipette filling solutions was S-ICS (pH_i=7.2), the cells were perfused with extracellular solutions having pH_e=8.0 (8.0-ECS, purple), pH_e=6.9 (6.9-ECS, blue), pH_e=7.4 (S-ECS, black), and S-ECS supplemented with 20 nM TRAM-34 (gray). The inset in panel A shows the KCa3.1-specific K^+ current measured below the activation threshold of Kv1.3, between −120 mV and −60 mV. Color coding of the traces is as in panel A. (**B**, **D**) KCa3.1-specific K^+ conductance (G_K) was determined by fitting straight lines to the traces in the inset in panel A for PBLs or to the straight lines in panel C (for CHO) and plotted as a function of time (B, same cell as in A; D: same cell as in C). The color of the symbols indicates the pH and/or TRAM-34 content of the solution.

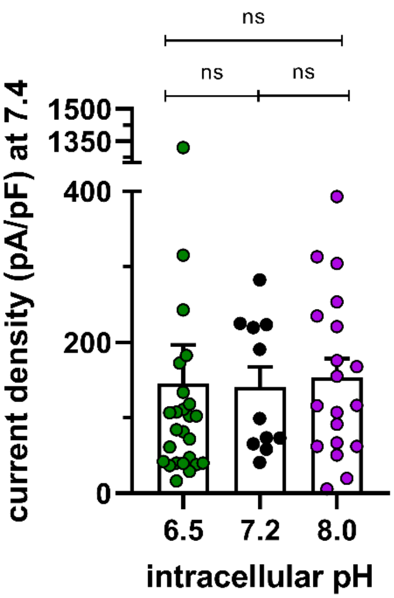

Supplementary Fig. 2. Current density of CHO cells transfected with turboGFP-hKCa3.1 at different intracellular pH. Bars and error bars indicate the mean ± SEM (6.5, green-filled circles: *n = 23*; 7.2, black-filled circles: *n = 14*; 8.0, purple-filled circles: *n = 18*) of the current densities of CHO cells transfected with turbo-GFP-hKCa3.1. At the beginning of each experiment with different intracellular pH the current at +50 mV was measured and then divided by the respective cell capacitance in order to obtain the current density.

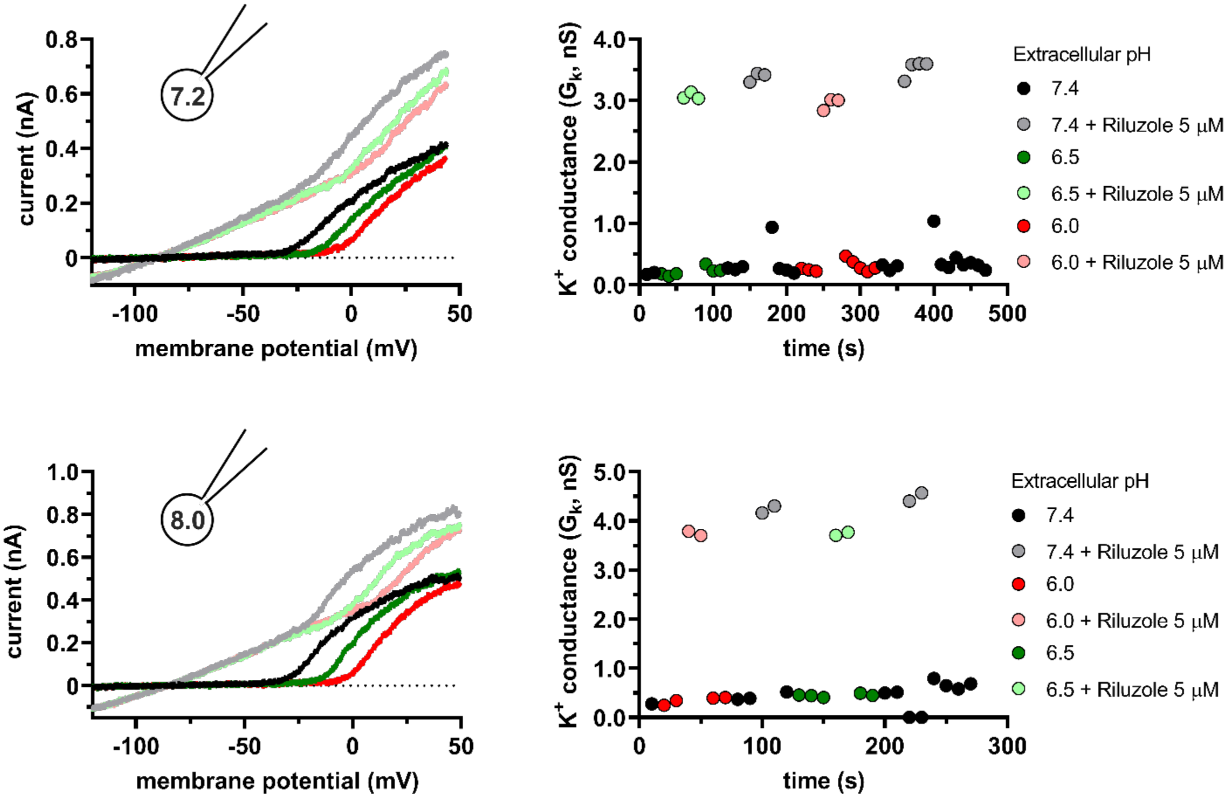

Supplementary Fig. 3. Effect of Riluzole on the KCa3.1 currents in PHA-activated PBLs at different pH_e and pH_i combinations. (**A**, **C**) Representative current traces were evoked by 150-ms-long voltage ramps, ranging from −120 to +50 mV in whole-cell patch-clamped human peripheral T cells. Voltage ramps were repeated every 10 seconds, the holding potential was −85 mV between pulses. The pipette filling solutions were 7.2-ICS-250 (pH_i=7.2, panels A and B) or 8.0-ICS-250 (pH_i=8.0, panels C and D). The cells were perfused with extracellular solutions having pH_e=6.0 (6.0-ECS, red), pH_e=6.5 (6.5-ECS, blue), pH_e=6.9 (6.9-ECS, green), pH_e=7.4 (S-ECS, black). The corresponding lighter colors display traces obtained in the presence of the modulator.
